# The Healthy Hearts Project: Development and evaluation of a website for cardiovascular risk assessment and visualisation and self-management through healthy lifestyle goal-setting

**DOI:** 10.1371/journal.pdig.0000395

**Published:** 2023-11-29

**Authors:** Imogen Rogers, Tom Grice-Jackson, Elizabeth Ford, John Howat, Remya Salimkumar, Kat Frere-Smith, Nicola O’Connor, Hilde Bastiaens, Harm van Marwijk

**Affiliations:** 1 Department of Primary Care and Public Health, Brighton and Sussex Medical School, Brighton, United Kingdom; 2 Brighton and Sussex Medical School, Brighton, United Kingdom; 3 Centre for Health Services Studies, University of Kent, Canterbury, United Kingdom; 4 William Joseph Ltd, London, United Kingdom; 5 Department of Primary and Interdisciplinary Care, Universiteit Antwerpen, Antwerp, Belgium; Iran University of Medical Sciences, IRAN (ISLAMIC REPUBLIC OF)

## Abstract

Materially deprived communities in the UK have excess morbidity and mortality from cardiovascular disease (CVD) but are less likely to engage with formal care pathways. Community engagement and e-health may be more effective ways to promote risk-reducing lifestyle change. The “Healthy Hearts Project” website was designed for use by community health workers (CHWs) for cardiovascular risk assessment and lifestyle goal setting, or for independent use by community members. This paper describes the website’s development and evaluation. The website was developed using interactive wire frame prototypes in a user-led approach. Qualitative evaluation of the completed website’s usability and acceptability was conducted using the “Thinking Aloud” method in a purposive sample of 10 participants (one voluntary sector employee, three CHWs, two community members and four healthcare professionals). Thinking Aloud interview transcripts were thematically analysed using an inductive approach. A separate quantitative evaluation of usability and the effect of using the website on CVD knowledge and beliefs was conducted. A random sample of 134 participants, recruited using the online platform Prolific, completed the “Attitudes and Beliefs About Cardiovascular Disease” (ABCD) questionnaire before and after using the website, along with the System Usability Scale (SUS). *Qualitative evaluation*—Four key themes were identified: 1) Website functionality and design—participants generally found the website easy to use and understood the risk communication graphics and the feedback and goal-setting features,; 2) Inclusivity and representation—most participants considered the website inclusive of a range of users/cultures; 3) Language and comprehension–participants found the language used easy to understand but suggested reducing the amount of text; 4) Motivation and barriers to change–participants liked the personalized feedback and empowerment offered by goal-setting but commented on the need for self-motivation. *Quantitative evaluation*–The mean score across all domains of the ABCD questionnaire (from 2.99 to 3.11, p<0.001) and in the sub-domains relating to attitudes and beliefs around healthy eating and exercise increased after using the website. The mean(sd) score on the SUS was 77.5 (13.5). The website’s usability was generally rated well by both quantitative and qualitative measures, and measures of CVD knowledge improved after use. A number of general recommendations for the design of eHealth behaviour change tools are made based on participants’ suggestions to improve the website.

## Author summary

Members of deprived communities in the UK are more likely to develop cardiovascular disease (CVD), but less likely to access health care services designed to reduce CVD risk. Community engagement and eHeath may be better ways to promote lifestyle change and reduce risk in these communities. We developed and assessed a website called the “Healthy Hearts Project” designed for community health workers (CHWs) to use with community members to help them assess their CVD risk and set healthy lifestyle goals. We interviewed a group of 10 health professionals, CHWs and community members while they used the website—most of them found the website easy to use and understand and liked the personalised feedback and the empowerment offered by the lifestyle goal setting. However, users suggested reducing the amount of text and commented that it required motivation to complete all the website stages. We also measured knowledge of CVD and the importance of healthy lifestyle before and after using the website in 134 participants using a questionnaire score, and found that it increased after using the website. Our participants’ suggestions on how to improve the website could be helpful for developing other eHealth tools that aim to encourage healthy lifestyle.

## Introduction

Cardiovascular diseases (CVD) are the leading cause of mortality worldwide [1] and account for 27% of deaths in the United Kingdom (UK). Research suggests that the majority of deaths from cardiovascular disease are preventable by changes to behavioural risk factors such as tobacco smoking, physical inactivity, poor diet and excessive alcohol consumption [2]. There is a marked social inequality in deaths from CVD in the UK, with those living in the most deprived areas being four times more likely to die prematurely of CVD than those in the least deprived areas [3]. This inequality is at least partly explained by social gradients in behavioural risk factors. For example, an analysis of the 2008 Health Survey for England data found that those with no qualifications were more than five times as likely as those with higher education to engage in four unhealthy behaviours [4]. Furthermore there is evidence that the impact of behavioural risk factors on cardiovascular mortality is greater among socioeconomically deprived groups [5].

As part of the UK’s CVD prevention strategy the National Health Service (NHS) Health Check Initiative was launched in 2009[6]. This is a universal screening programme, where attendees are offered a 10-year CVD risk assessment, with those estimated to have a 10% or higher risk of a cardiovascular disease or event in the next ten years being offered a lifestyle consultation and medication if appropriate. However, modelling suggests that such universal screening programmes have little effect on health inequalities and may even exacerbate them, with uptake of health checks being poor in socioeconomically disadvantaged areas. Difficulty in accessing a General Practitioner (GP—or primary care physician) has been highlighted as a common barrier to attending health checks, and recent research has shown that socioeconomically deprived areas have been worst hit by shortages of GPs, and that this trend appears to be worsening over time [7].

An alternative way to ‘deliver’ CVD prevention interventions is via community engagement, which has been described as “the process of working collaboratively with and through groups of people affiliated by geographic proximity, special interest, or similar situations to address issues affecting the well-being of those people.” Community-engagement approaches can make use of trained non-healthcare professionals to provide screening and health promotion activities in the community, potentially reaching individuals who are not captured by formal care pathways. Community health workers (CHWs) can deliver culturally acceptable and understandable health information. This process can be facilitated by access to appropriate eHealth/mHealth tools, with evidence that that the use of these tools to support CHWs can help improve health outcomes in a number of areas including maternal health, infectious disease prevention and cardiovascular health [8,9].

The Scaling-up Packages of Interventions for Cardiovascular disease prevention in Europe and Sub-Saharan Africa (SPICES) project aimed to co-design and implement a community-engagement motivational lifestyle coaching intervention for individuals from materially deprived communities in the South-east of England. This was delivered by trained volunteer community health workers (CHWs), based within local partner voluntary and community and social enterprises (VCSEs). The aim was to help people to improve their lifestyle and reduce cardiovascular disease risk by increasing physical activity, decreasing intakes of fat, salt and sugar, increasing intakes of fibre, oily fish and fruit and vegetables, and decreasing levels of cigarette smoking. The SPICES programme has been described in more detail elsewhere [10]. The coaching was informed by the results of a number of questionnaires completed by the participants which aimed to assess current CVD risk, and behavioural risk factors for CVD, i.e. smoking, dietary intakes and physical activity levels.

One of the aims of the project was to develop a website that could be used as a screening and goal-setting tool by the CHWs or used alone by community members using the content of the screening questionnaires administered in the SPICES study. The website was intended to be used both for assessment of current cardiovascular risk in order to identify a moderate risk population suitable for inclusion in the intervention, for evaluation of the behavioural risk factors and to assist in the setting of healthy lifestyle goals. This paper describes the development and evaluation of the website. Two types of evaluation were performed on the website: 1) a qualitative evaluation to evaluate key stakeholders’ perceptions of the website and its suitability for the target audience, and to develop recommendations for improvement and 2) a quantitative evaluation to assess the website’s usability and effect on attitudes and beliefs toward CVD.

## Methods

### Website development tools and methodology

**Fig 1:**
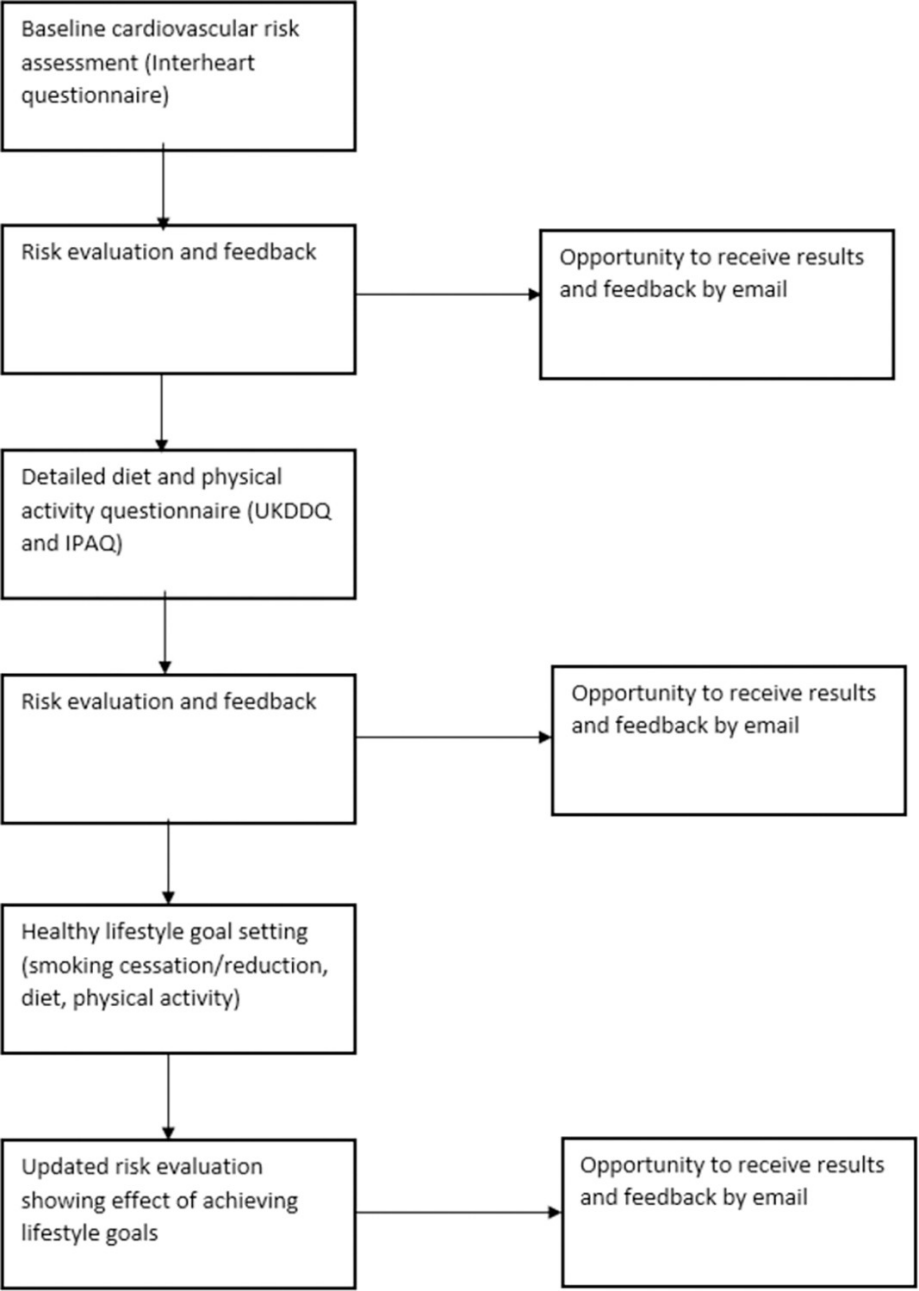
User journey through the website.

The “Healthy Hearts Project” website (www.healthyheartsproject.co.uk) was produced by William Joseph Ltd [11] with input from the research team over a period of 12 months. A schematic representation of the user journey through the website is given in Fig 1. Information on the intended users’ perspectives on CHD and lifestyle behaviour change derived from interviews and focus groups conducted as part of the pre-implementation phase of the SPICES project [12], was taken into consideration in the website’s design and language.

Given the complexity of the proposed website, and the number of questions users needed to answer, the entire journey was prototyped using interactive wireframes (basic representations of website design which allow the user to click through and test the navigation and interactive elements) in a user-led approach and a design-phase Thinking Aloud assessments [13] was conducted with 13 individuals from the target user group and members of the SPICES consortium over two rounds of testing. A “Thinking Aloud” protocol is a semi-structured interview style where the participant is encouraged to verbalise their thoughts as they work through a website or application [13,14]. (The design-phase Thinking Aloud discussion guides used are provided in S1 Appendix and S2 Appendix). Feedback from both rounds was incorporated iteratively to develop the final product. Testing focused on both product features and language, and improvements were prioritised based on maximising benefit to the user.

### Website evaluation methods

Two types of evaluation were conducted on the completed website–a qualitative “Thinking Aloud” study (this was in addition to the separate “Thinking Aloud” assessment conducted during the design phase, the discussion guides are given in S3 Appendix) and a quantitative questionnaire-based evaluation of the website’s effect on CVD knowledge and attitudes.

### 1) Qualitative Evaluation

**Participants:** Participants for the qualitative evaluation were recruited via links to the SPICES project, partner VCSEs and research team and fell into four categories: 1) Healthcare professionals (HP) including general practitioners and NHS commissioners 2) Key Individuals (KI) in leadership roles in the partner VCSEs and 3) CHW volunteers participating in the SPICES project 4) Community member (CM) participants who had received the SPICES intervention. Participants were emailed an invitation to the study along with the participant information sheet, consent form, and a short participant details questionnaire.

**Research instrument and data collection:** Individual interviews following a “Thinking Aloud” protocol and lasting approximately one hour were conducted via video-conferencing using Zoom software. A discussion guide was used, which attempted to elicit the participants’ understanding of, expectations from, and emotional responses to each stage of the Healthy Hearts Project website. Two researchers conducted each interview, with one (RS) acting as the main interviewer and the other (JH or TGJ) taking notes. We aimed to recruit at least 10 participants based on the results of a meta-analysis by Hwang et al.[15] which indicated that 10 +- 2 participants is the optimal sample size for detecting usability problems using the “Thinking Aloud” method.

**Data analysis**: Interviews were recorded and automatically transcribed by Zoom, transcripts were reviewed with the recording and edited for accuracy by RS. All identifying information was removed from interview transcripts prior to analysis. Thematic analysis of qualitative data was carried out in NVivo by RS using an inductive approach. Line-by-line first level coding was initially conducted on information pertaining to the research aims. These codes were then arranged into groups of meaningful concepts that made up the second level codes. These secondary codes were reduced to the minimum number of themes that adequately described all the data. Themes were reviewed and edited by two other researchers (TGJ and IR) until consensus was reached. The final themes were used to summarise participant perspectives on the website and to develop key recommendations for its improvement.

### 2) Quantitative Evaluation

**Participants:** This evaluation recruited participants via Prolific [16], a platform for online subject recruitment catering explicitly to researchers. Prolific has a number of advantages over other recruitment platform such as Amazon’s Mechanical Turk, including a greater naivety of subjects to online surveys and a more diverse subject pool [17]. It also allows pre-screening of participants based on a number of socio-demographic characteristics including age and educational level. In this study UK-resident participants aged 30y or over whose highest educational qualification was less than undergraduate degree level were selected in order to be more representative of the target audience for the website (7495 Prolific participants met these criteria on 15^th^ May 2023). Participants were paid at an hourly rate of £8.72 for their participation in the survey. A random sample of eligible subjects registered on the Prolific platform who fit the recruitment criteria were sent an email invitation to the study survey, recruitment continued until the target number of participants had been reached, some additional participants over the target were then recruited until funding for this aspect of the study was exhausted.

**Research instruments and data collection:** Participants’ attitudes surrounding CVD and modifiable risk factors before and after using the website were assessed using a modified version of the Attitudes and Beliefs about Cardiovascular Disease (ABCD) survey [18], a short validated questionnaire designed to assess awareness of CVD risk on the basis of knowledge, perceptions of CVD and intention to change behaviour. This includes 20 four-point Likert Scale questions designed to assess 4 key domains: perceived risk of heart attack or stroke, perceived benefits of and intention to exercise, perceived benefits of and intention towards healthy eating, and benefits of and intentions to stop smoking, Cronbach’s alpha for these four subscales in a recent validation study were 0.86, 0.84, 0.84 and 0.85 respectively [18]. This questionnaire was selected as although a number of validated questionnaires are available to measure CVD knowledge, perceptions, or intention to change behaviour [19–21], the ABCD is the only short validated questionnaire to measure all these aspects of awareness of CVD risk. Participants were asked to complete the ABCD questionnaire both before and immediately after completing all sections of the Healthy Hearts Project website. Each question was scored from 1 to 4, with 4 indicating the highest CVD knowledge or intention to change behaviour, and 1 the lowest. A mean answer score was calculated across the whole questionnaire (total score), and within each of the 4 domains. For those participants who did not smoke, the mean total score was calculated excluding the questions on smoking.

Website usability was evaluated using the System Usability Scale [22], a widely-used 10-item scale for assessing the usability of websites and applications, including e-health tools [23] (a copy of the SUS is given in S4 Appendix). Answers to the SUS questionnaire were used to calculate a score out of 100, with higher scores indicating better usability. SUS was presented to participants after they had used the Healthy Hearts Project website. The time taken to complete the whole website was also recorded.

**Data analysis:** For the questionnaire-based part of the study we aimed to recruit 100 participants. With a power of 80% and a two-sided alpha = 0.05, a sample size of 100 would enable detection of an effect size (Cohen’s d) of 0.285 on pre-post change to measures of CVD knowledge or intention to change behaviour from the ABCD questionnaire (with 0.2 being considered a small effect, and 0.5 a medium effect). Mean scores across the ABCD (mean total score) and for each of the sub-domains before and after completing the intervention were compared using paired t tests. Multiple linear regression was used to investigate how change in mean total score was associated with age, sex, pre-test score and time spent completing the website (dichotomised as over or under 20 minutes). All analyses were conducted using IBM SPSS Statistics 26.

### Ethical approval

This study received ethical approval from the BSMS Research Governance and Ethics Committee (RGEC) (reference numbers ER/BSMS8944/1 and ER/BSMS99A4/1). All participants signed an informed consent form prior to taking part.

## Results

### Website content and structure

The website led users through the questionnaires described below which aimed to assess current CVD risk and behavioural risk factors for CVD.

**Interheart questionnaire:** Baseline cardiovascular risk assessment was conducted using the validated short non-clinical Interheart questionnaire [24]. This includes questions on age, sex and family history, presence of high blood pressure and diabetes, psychosocial factors (stress and depression), smoking and passive smoking, intakes of fruit, vegetables, salty foods, fatty food and meat and poultry, and physical activity levels. This questionnaire was scored to give a total cardiovascular risk score between 0 and 48 with 0–9 being considered low risk, 10–15 moderate risk and 16–48 high risk [25]. (Details of scoring given in S5 Appendix).

**UKDDQ and IPAQ:** To evaluate conformity to healthy eating guidelines and assist in the setting of healthy eating guidelines, diet was assessed using a brief 21-item food frequency questionnaire based on a modified version of the UK Diet and Diabetes Questionnaire (UKDDQ)[26]. This questionnaire was used to derive a diet quality score between 0 and 80 based on reported consumption of fruit, vegetables, wholegrains, meat, fish and fatty, sugary and salty foods. Higher scores were indicative of a “healthier diet” with scores of 55 or over considered to be in the healthy/green range, scores of 40-<55 in the moderate or amber range, and scores of <40 in the unhealthy/red range. Physical activity levels were measured using the International Physical Activity Questionnaire (IPAQ)[27,28]. The IPAQ is an internationally validated instrument to capture information about weekly physical activity habits, behaviours, and routines [29]. The answers to the IPAQ were used to calculate an activity level in terms of metabolic equivalents (MET scores) or number of minutes spent in moderate of vigorous activity which was then classified as either inactive, moderate or high activity level. Details of the IPAQ scoring are given in S6 Appendix, details of the UKDDQ scoring are available from the authors on request.

**Fig 2:**
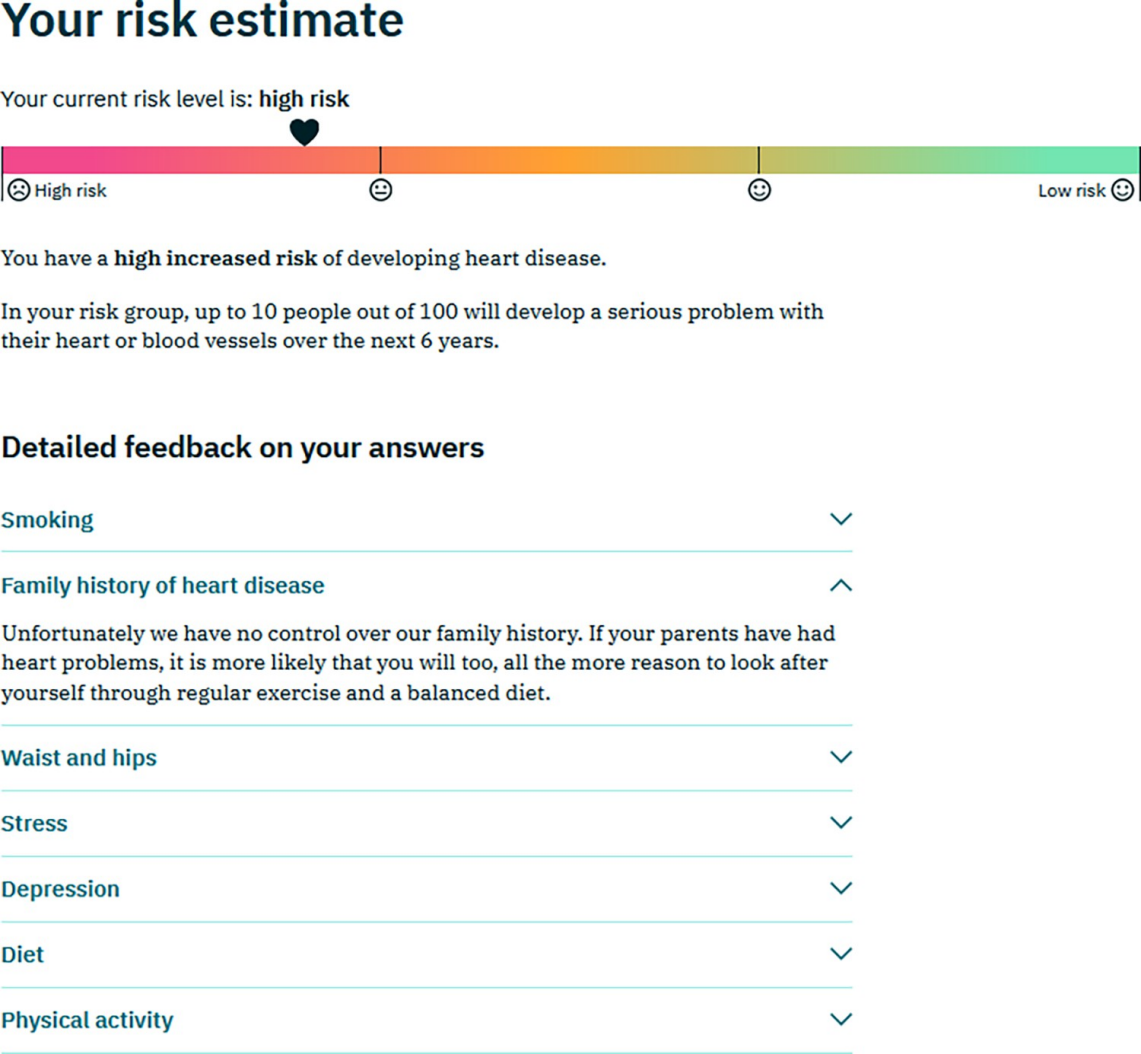
Example of risk feedback after completion of the Interheart Questionnaire.

**Baseline risk assessment and feedback:** The user was asked to complete the baseline cardiovascular risk assessment (Interheart)questionnaire. They were then presented with both a visual representation on a red-amber-green (RAG) scale and a text-based summary of their cardiovascular risk, along with a number of drop-down items with more detailed information on different risk factors (Fig 2). Although alcohol consumption was not included in the baseline risk assessment (this information was collected later as part of the modified UKDDQ) a decision was taken to provide generic advice on alcohol consumption at this stage in the initial feedback to all users. This was because users may choose to quit the website at this stage and it is an important lifestyle determinant of CVD risk.

**Fig 3:**
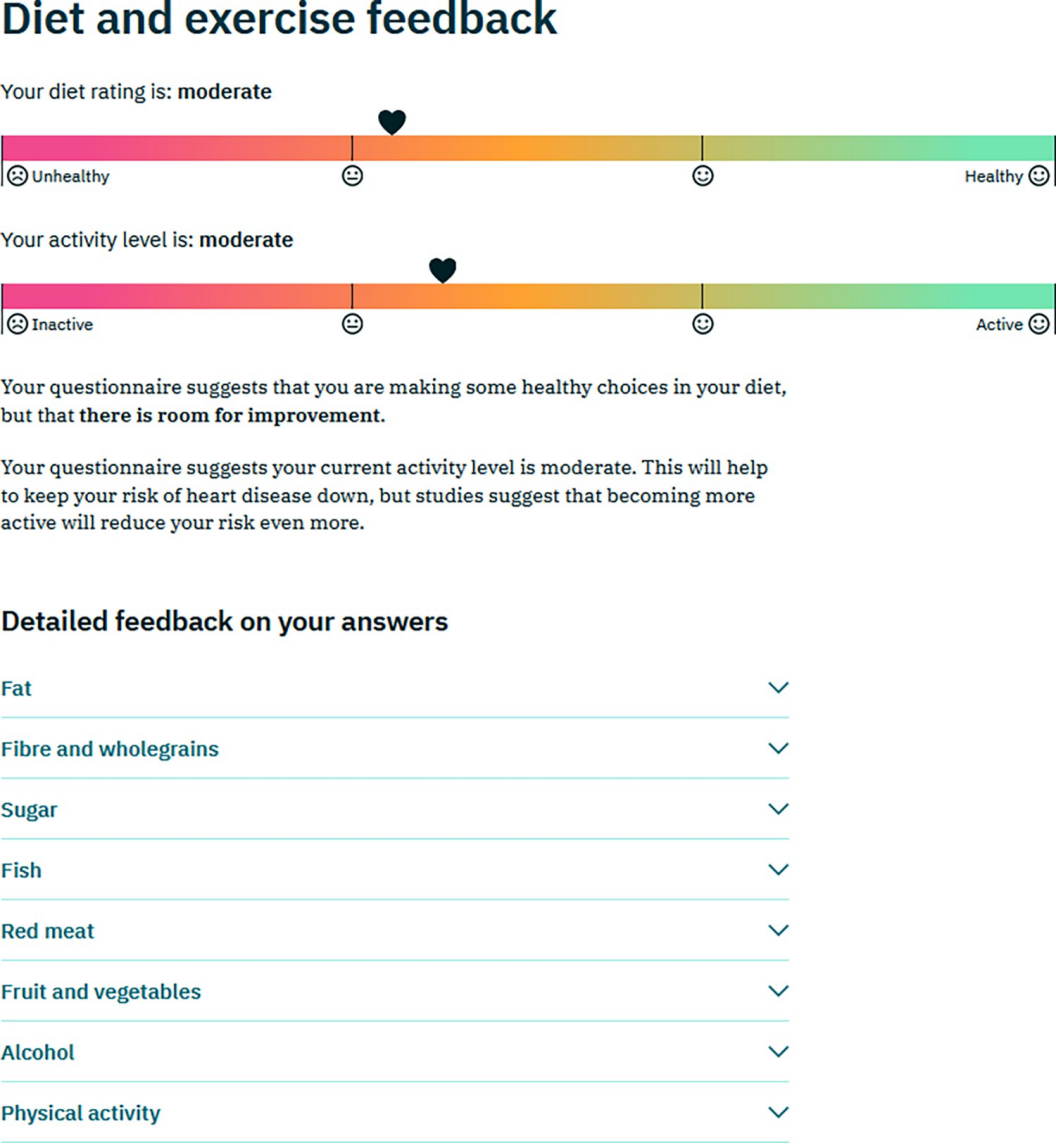
Example of feedback after completion of the diet and physical activity questionnaire.

**Detailed assessment of diet and physical activity levels:** The user was then invited to complete the more detailed questionnaires on diet, alcohol drinking, and physical activity level (UKDDQ and IPAQ), after which they were given feedback on how their answers compared to recommendations. Again a visual representation on a RAG scale and a text-based summary was provided, along with drop-down items with more detailed feedback (Fig 3).

**Fig 4:**
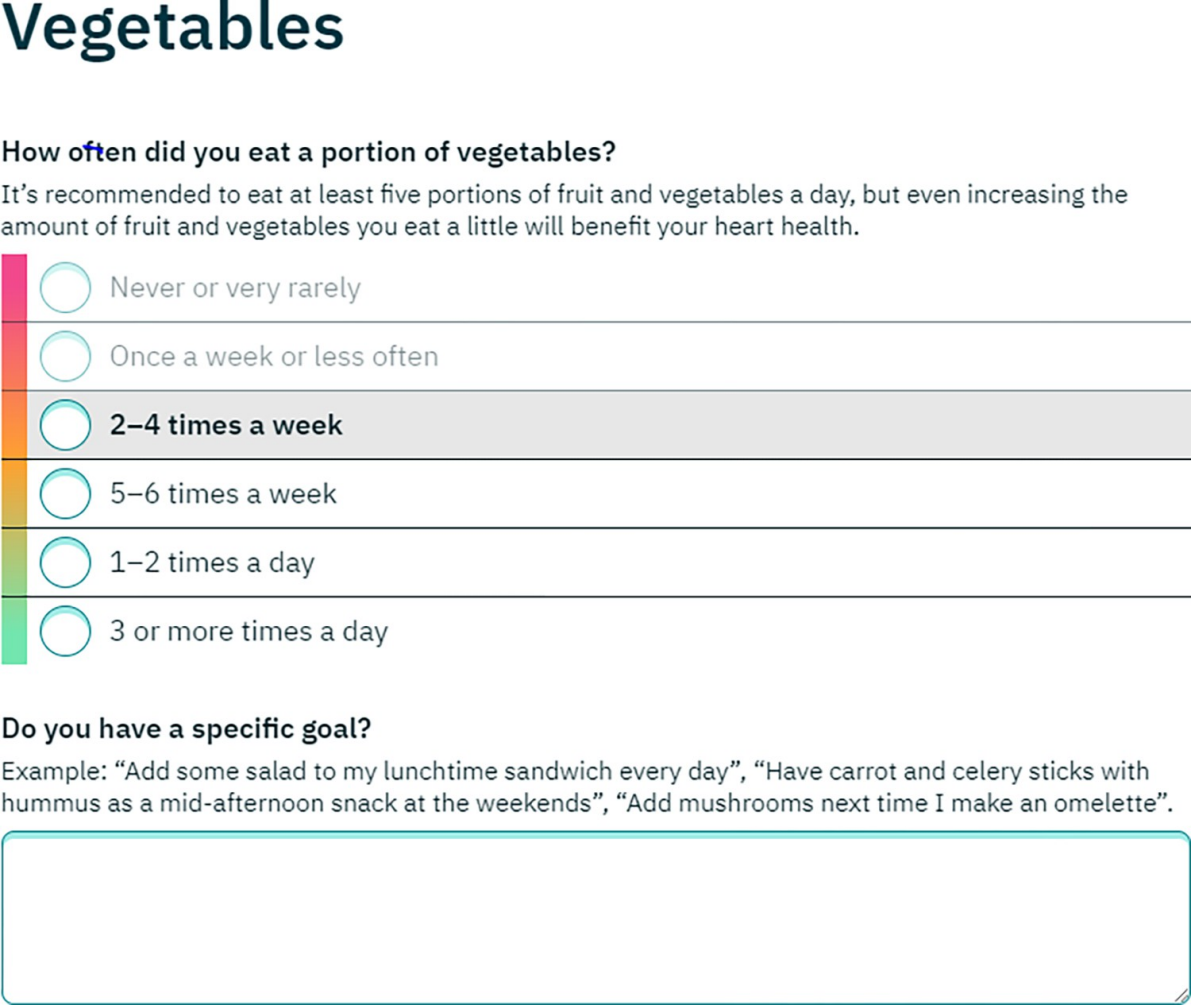
Example of a dietary goal setting page.

**Fig 5:**
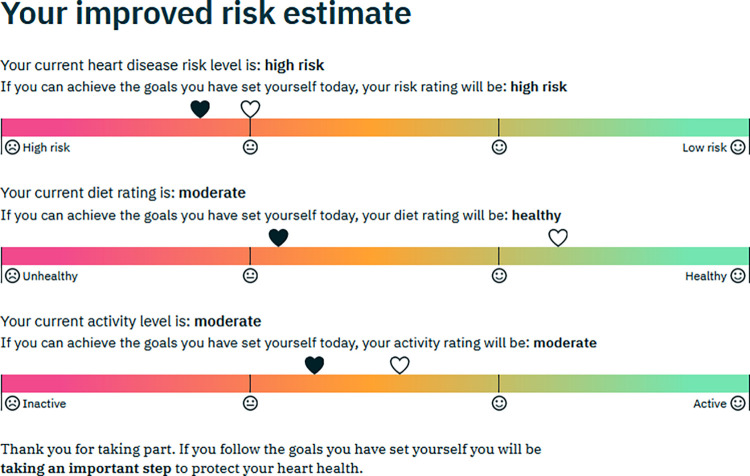
Example of the final feedback page.

**Healthy lifestyle goal setting:** After both questionnaires had been completed there was an opportunity for healthy lifestyle goal setting for 1) smoking reduction or cessation 2) diet 3) physical activity. The section worked by repeating the relevant questions in the Interheart, UKDDQ and IPAQ questionnaires–the user’s original answer was highlighted, and they were invited to select a “healthier” answer as their goal (Fig 4). The goal setting section was customised according to the user’s answers to the questionnaires. For example, for an option to set a goal for smoking reduction/cessation was only offered to those who reported being active smokers. In the section on diet, options to set goals were only offered for those foods where the original questionnaire suggested less “healthy” current levels of consumption, e.g. an option to set a goal to reduce intake of cakes and biscuits (cookies) would be offered to those who reported consuming these items twice a week or more, but not to those who reported consuming them once a week or less. The user was asked to select a target level of consumption of each food, and was encouraged to set a “SMART” goal [30] by describing in a text box exactly how they would make the dietary changes. In the physical activity goal setting section, the user was invited to set a goal for increasing time spent walking or engaging in moderate and vigorous physical activity, and again there was an option to create a “SMART” goal using a text box. On completion of the goal setting section there was a final feedback page where the user was shown how achieving their healthy lifestyle goals would affect their overall cardiovascular risk, and an updated assessment of their diet and physical activity levels was presented. (Fig 5).

**Option to receive results by email:** After completion of each questionnaire and the goal setting section the user was offered the opportunity to enter their email address to receive a copy of their questionnaire answers, any goals set, and feedback given up to that stage.

**Data storage**: No data was stored from a user interaction with the website. This decision was made so that no data protection laws had to be considered or risk assessed. As no data was stored the decision was also taken not to include hyperlinks in the website, as depending on the browser this might result in users navigating away from the website and losing their progress.

**Basis of recommendations given in feedback text:** All introductory and feedback text was written to accord with current UK NHS recommendations on diet, exercise, waist:hip ratio and alcohol consumption [31,32], and was reviewed by a UK qualified Health Coach (KFS) who formed part of the research team.

#### Improvements made to the website as a result of the design phase Thinking Aloud assessments

These improvements included:

- simplifying academic language and terminology and using plain language to make the website easier to understand.
- focusing on intuitive design throughout so that users could finish all of the tasks with a high success rate, for example by using buttons and sliders and always ensuring a simple way to navigate back to the previous screen.
- adding instructional information at each stage so that users knew what to expect, and felt confident and prepared for the tasks ahead of them.
- giving participants a simple top-line visualisation to show their progress through the tool and their overall score.
- improving accessibility for visually-impaired users by enhancing the established red-to-green ‘traffic light’ system with emoji faces.
- designing an achievable goal-setting experience to increase the likelihood that people will make small changes that stick.

### Qualitative results

Ten individuals were interviewed as part of the qualitative Thinking Aloud evaluation of the completed website (55 were initially invited to participate, 3 declined and 42 did not respond to the initial or follow up emails). The characteristics of the 10 participants are summarized in Table 1.

**Table 1:** Characteristics of individuals taking part in the qualitative “Thinking Aloud” evaluation.

Characteristics	Category	n
**Sex**	Female	7
	Male	3
**Age**	18-35y	4
	36-54y	2
	55+	4
**Ethnicity**	White British/Irish	8
	South Asian	2
**Highest Educational Level**	Postgraduate Degree	6
	Degree	3
	2 or more A Levels or equivalent	1
**Role**	Healthcare Professional (HP)	4
	Voluntary Sector Key Individual (KI)	1
	Community Health Worker Volunteer (CHW)	3
	Community Member (CM)	2

**Fig 6:**
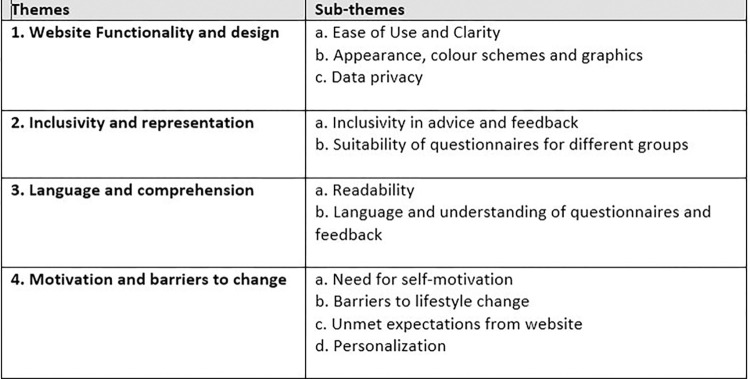
Themes and sub-themes relating to perceptions of the website and its suitability for the target audience.

Four key themes were identified in the data: 1) Website functionality and design 2) Inclusivity and representation 3) Language and comprehension 4) Motivation and barriers to change. These themes and their subthemes are shown in Fig 6.

#### Theme 1 Website Functionality and design (illustrative quotes provided in Table 2)

**Table 2:** Illustrative quotes for Theme 1: Website functionality and design.

**1.1 Ease of use and clarity**
*“The layout is very clear*, *it’s very simple*, *very easy to read … I found it kind of guided you through … and I totally understood the reasons for it asking the questions it did*.*”* CM 1
*“I like the videos*, *I think that’s absolutely fantastic*, *I like the way that there is a clear easy instruction for both sort of people that understand things through reading as well as kind of that more audio visual learning*.*”* HP1.
*“Setting achievable goals*, *it’s good to say that*, *and specific*, *it’s the SMART goal thing*, *isn’t it*? KI1
**1.2 Appearance colour scheme and graphics**
*“I wasn’t expecting the green … NHS has a blue and white logo and many other organisations that I work with have a blue and white logo”* HP1”
*“I like the scale of the faces and the rainbow colours; it’s nice and engaging and everything*, *and the little heart on it*, *so although obviously it’s reflecting medical kind of scorings behind the scenes it’s nice and light and accessible”* KI1
*“I would just want to sense check that scale of sad face to happy face*. *I don’t know if it’s needed*, *umm*, *is there some other icon or imagery which would feel less sort of approving versus disapproving in a scale*.*”* HP1
**1.3 Data privacy**
*“the safety of their personal data*, *probably the most important thing initially*, *and people will then self-select in or out*. *If they’re interested in their heart health they’ll select in*.*”* KI1

### 1.1 Ease of use and clarity

All participants understood the purpose of the website from a brief glance at the landing page, and with a longer look most had a clear idea of the different steps involved, although one participant did not realise that multiple questionnaires were involved. Most participants noted the statements on how long it would take to complete the different stages, with one commenting it was important to “manage expectations” about this. Most were clear about the need for a tape measure (for measurement of waist and hip circumference) for the Interheart survey. Several participants commented positively on the use of a Youtube video embedded in the Interheart questionnaire to demonstrate how to take waist and hip measurement. The purpose of the goal setting section was generally well-understood and one participant commented that they liked the fact that you could skip goals if you wanted to and that goals were designed to be specific and achievable. Most participants understood the purpose of the text box to add a SMART goal description, although one was unclear about this.

A number of features caused confusion among several participants. 1) Saving progress–the landing page included a statement under the heading “Can I save my progress” that progress would be lost on closing the browser or leaving the site. However, this was poorly understood by the participants, several of whom assumed from the heading that progress could be saved. 2) As the Interheart questionnaire includes a number of questions on diet, several participants questioned the need to assess diet again using the UKDDQ. 3) The mechanism to set number of days of physical activity performed confused participants, who expected to be able to be able to “slide” this up from 0, instead of needing to click on the correct number of days. 4) In the goal setting section several participants were not clear that the highlighted option represented their previous answer.

### 1.2 Appearance, colour schemes and graphics

Participants had mixed views on the website’s colour scheme (example given in S1 Fig). One commented several times that the website should use more colour, another felt the colour scheme was “friendly” and “attractive and gentle”, a third felt it lacked a “medical feel” as it did not mirror the blue and white colour scheme used by the NHS.

Participants generally commented positively on the various graphics given to represent risk (Figs 2, 3 and 5). Most participants liked the RAG colour scheme and the use of smiley/sad faces to indicate low/high risk, However, one felt that the smiley/sad faces might be perceived as judgemental, and another expressed a preference for “numbers and percentages” rather than a graphic. Several participants said they liked the graphics giving the risk feedback after goal setting (Fig 5). It was generally felt to be useful to see all the risk measurements together, and that the potential improvement in risk of reaching the goals was shown in an “accessible way” and offered “positive reinforcement”. However, three participants were unclear about the meaning of the two heart icons representing pre- and post-goal risk.

### 1.3 Data Privacy

Six participants commented on data privacy, with five noting that personal data was not collected, and several commenting on this positively. One suggested that personal data safety was “probably the most important thing initially” in a user’s decision whether to engage with the website. One participant believed that their personal data would be collected, and commented that they wanted more information on how it would be kept secure.

#### Theme 2 Inclusivity and representation (illustrative quotes provided in Table 3)

**Table 3:** Illustrative quotes for Theme 2 Inclusivity and representation.

**2.1 Inclusivity in advice and feedback**
*“You know a lot of heart disease patients*, *you know may or may not be just talking about ethnic groups*, *where you know it’s more prevalent*, *is it available in different kind of languages or translated*?*”* HP4
*“That’s nice*, *chair exercise*, *that’s nice to have that in that’s very inclusive*, *acknowledging the fact that not everyone is going to be able to cycle or whatever*.*”* KI2
*“I’m just wondering if there’s an application in schools because that’s one of the things that you know they’re talking about*: *healthy diets*. *And something that would be tailored to children…”* CM1
**2.2 Suitability of questionnaires for different groups**
*“I’m a vegetarian*, *I don’t eat fish*, *I do supplement with omega oils*, *so I sort of felt like when I was filling those in… the website might interpret my risk in a certain way because I couldn’t put any mitigating factors in”* CHW2
*“if you’re digging holes in the ground day in*, *day out … it’s probably worth having something about occupation there as well*.*”* CHW3

### 2.1 Inclusivity in advice and feedback

Three participants (KI2 and HP2 and HP4) noted that the examples given for physical activity in the goal setting section were inclusive of older people and the disabled, with one (HP2) suggesting that perhaps the issue of disability should be raised earlier in the website journey. One (HP4) raised the issue that heart disease is more prevalent in some ethnic groups, and asked if the website could be made available in languages other than English. Another commented that presenting risk information via the graphics rather than just text made this more accessible to users whose first language was not English. Two participants commented that the website could be made more inclusive of younger people and that the graphics depicted mostly older adults which might make it harder for young people to relate to. It was also suggested that the tool could be useful for children given the increasing rates of child obesity.

### 2.2 Suitability of questionnaires for different groups

Four participants commented on the question on sex at the beginning of the Interheart questionnaire, with 3 stating that it was not inclusive of trans and non-binary individuals, and one suggesting it should be made clearer the question referred to “biological sex”. It was also suggested the Interheart should include questions on ethnic group, activity at work to cover those with manual jobs, and presence of “pre-diabetes”. Several participants praised the range of examples given in the UKDDQ, which were felt to be inclusive of a range of incomes, lifestyles and cultures. Conversely, other participants felt that the questionnaire would need to be expanded to reflect their diets, in particular to capture gluten and dairy free foods and reflect vegan and vegetarian diet and supplement use.

#### Theme 3 Language and comprehension (illustrative quotes provided in Table 4)

**Table 4:** Illustrative quotes for Theme 3: Language and comprehension.

**3.1 Readability**
*“If I was reading this as somebody who had issues with literacy I’d already be struggling with it… I’m just thinking about the average reading age in this country is nine or so… Already that’s too much information to take in…it’s quite kind of overwhelming*.*”* HP2.
*“Just highlighting maybe what they can do*, *or you might want to replace some of the text to links to kind of you know NHS choices*, *or you know patient*.*co*.*uk*, *which gives further information that they can look up*.*”* HP4
*“It’s about how we can perhaps condense words down to kind of key statements that are separated a bit*, *there’s a lot you’d have to read that even though it’s not huge amount of text it’s still how its set out*.*”* HP2
**3.2 Language and understanding of questionnaires and feedback**
*“I train every day and I run but that’s not my leisure time…in my leisure time I’m not doing anything*, *I’m chilling*.*”* CM2.
*“I like how they’ve got lots of different examples under each heading*, *helps kind of people put their food in categories*, *that’s very clear*.*”* HP3
*“I’m not sure quite what vigorous activity means because I don’t jog but I do walk quickly and I garden a lot*.*”* CM1

### 3.1 Readability

The majority of participants commented that there was too much text throughout the website, particularly on the questionnaire landing and feedback pages. It was suggested that given the relatively low average reading age in the UK that this might make the information hard to absorb for some users, particularly those in the target demographic from materially deprived communities. A number of strategies were suggested by participants to deal with this. These included using bullet points rather than blocks of text, highlighting key messages, and replacing some of the text with links to external sources of information.

### 3.2 Language and understanding of questionnaires and feedback

Several participants suggested more colloquial language could be used in the Interheart questionnaire, and questioned whether users would know if they had conditions such as high blood pressure or suffered from stress, or understand a number of terms used e.g. “passive smoking” or “leisure-time physical activity”. There were also a number of comments on the Interheart feedback. Several participants questioned why advice on alcohol consumption was given as this was not covered in the questionnaire. It was suggested that information on waist:hip ratio (WHR) ranges representing low, medium and high risk could be given as well as the user’s WHR and risk level It was also suggested some information given in the feedback could be incorporated into the questionnaire to clarify why questions were being asked, e.g. some brief information on the relevance of diet, physical activity, stress and WHR to CVD risk.

Participants generally found the UKDDQ questions easy to understand and answer, and liked the specificity and range of examples given. Four participants suggested adding a graphic to illustrate alcohol units to the question on drinking. Most participants thought the instructions for the IPAQ were understandable, and liked the examples given, but several said the distinction between moderate and vigorous activity could be subjective. The feedback after the diet and exercise questionnaires was generally felt to be easy to understand, with one participant praising the suggestions for “straightforward changes that would make a bit difference”. Suggested changes to the feedback included more specific suggestions on how to increase activity levels.

#### Theme 4. Motivation and Barriers to Change (illustrative quotes provided in Table 5)

**Table 5:** Illustrative quotes for Theme 4: Motivation and barriers to change.

**4.1 Need for self-motivation**
“It’s quite cumbersome, so I think you need to be pretty motivated, you need to have a fairly good reading age” CHW3.
*“That’s really nice motivating positive facts*, *that you know within a few hours of stopping smoking it’s already started clearing from your blood… that gives people a bit of hope… I like that*.*”* KI1
*“if I am following my goals*, *yeah*, *I think that’s quite good actually because if I had been in like the orange zone or the red zone and I set some goals*, *I could see my heart moving into a better area*, *say into a green area*.*”* CHW2
**4.2 Barriers to lifestyle change**
*“Yeah it’s not asking them for any info around their financial*, *you know how food access*, *food poverty*, *digital stuff … mental health and well-being*, *social isolation*, *digital isolation*, *their sense of connection with their community or their friends and family*” KI1
*“They know what they should be doing … to improve their health*. *What they’re looking for is support really rather than hard facts and information*.*”* CHW2
**4.3 Unmet expectations from website**
*“I wonder if it might be interesting to have a line or two maybe at the start of the questionnaire saying … like all the information included is from a legitimate source like maybe a line about the research team and their credentials … just a line to make you feel very confident that you’re looking at credible sources of information*.*”* CHW1
**4.4 Personalization**
*“Getting the depth and quality of the risk assessment and the risk factors in there to do that effectively is the trade off of doing something short and sweet*, *but it does feel personal and it does feel tailored to me*, *which I think is good*.*”* HP1

### 4.1 Motivation and empowerment

Several participants commented that users would need to have quite high levels of self-motivation in order to set and follow goals using the website. Two questioned whether users who were low risk on the Interheart would have any incentive to proceed to the later stages of the website. One felt that for potential users to engage, it would need to be introduced in an “engaging way within kind of one to one conversations” rather than e.g. via email, but suggested that “once people are in the door and their interest is piqued” they would be likely to go on and complete the goal setting. Participants did pick out a number of features of the website they considered motivating. Several commented that the process of filling in the diet questionnaire acted as a prompt to reassess their dietary choices, with one describing it as “awakening”. The ability to set goals independently was felt to be empowering. Two participants said that the graphics showing the potential risk improvement on achieving lifestyle goals would act as a motivator to change, and showed the room for improvement even for those with relatively healthy lifestyles. Several commented on the “reassuring” and “positive” tone of the feedback offered, although others stressed the need to avoid messages which might be perceived as judgemental or “alarming”.

### 4.2 Barriers to lifestyle change

Several participants discussed the broader determinants of cardiovascular disease, such as poverty, mental health problems and social isolation, and pointed out that a website could not address these issues. The issue of digital exclusion among materially deprived communities was raised. Two highlighted the need for support to make lifestyle changes as well as just information, and one suggested adding links to services offering support with behaviour change.

### 4.3 Personalization

Three participants commented positively on the “personalized” feel of the website and the feedback offered. One said that while the website took some time to complete, the “tailored” advice given as a result was the “trade-off” for this. However, one participant questioned why they were being asked to set a goal for vigorous activity when their activity level was already high, and said this section felt “not personalized and not tailored”.

### 4.4 Unmet expectations from website

One participant suggested adding information on how many people had benefited from using the website, and another advised highlighting the credentials of the research team contributing to the website on the landing page. In line with this some participants felt that the advice given on the website contradicted that received from other sources perceived to be more authoritative, e.g. one questioned being given a “moderate” activity rating when they were exercising in line with British Heart Foundation guidelines. Two participants suggested including a prompt for users to visit their GP about any concerns. There were also a number of comments on how to improve the goal setting section, including being able to go back and modify goals after viewing their effect on risk estimates, and the option of skipping straight to physical activity goals for those with a “healthy” diet rating.

### Quantitative results

146 people attempted the survey, of whom 12 did not complete it giving a final sample size of 134. Participant characteristics are summarized in Table 6.

**Table 6:** Summary of participant characteristics.

Gender	Male	42 (31.3%)
	Female	92 (68.7%)
**Age (y)**	Mean (SD)	40.87 (9.55)
	30–39	77 (57.5%)
	40–49	31 (23.1%)
	50–59	19 (14.2%)
	60–69	7 (5.2%)
**Highest educational level**	No formal qualifications	4 (3.0%)
	Secondary education	32 (23.9%)
	Technical/community college	38 (28.4%)
	High-school diploma/A level	60 (44.8%)
**Employment status**	Employed full time	64 (47.8%)
	Employed part time	28 (20.9%)
	Not in paid work (e.g. homemaker, retired, disabled)	23 (17.2%)
	Unemployed and job seeking	7 (5.2%)
	Other	2 (1.5%)
	Missing	10 (7.5%)
**First language**	English	124 (92.5%)
	Other languages	10 (7.5%)
**Country of Birth**	Brazil	2 (1.5%)
	Bulgaria	1 (0.7%)
	Czech Republic	1 (0.7%)
	Germany	2 (1.5%)
	Iran	1 (0.7%)
	Nigeria	1 (0.7%)
	Pakistan	1 (0.7%)
	Portugal	1 (0.7%)
	United Kingdom	123 (91.8%)
	Missing	1 (0.7%)

The results of the ABCD survey before and after completing the website are summarized in Table 7.

**Table 7:** Mean scores in each domain of the ABCD questionnaire before and after completing the Healthy Hearts Project Website.

Domain	Pre-test score Mean (SD)	Post -test score Mean (SD)	Change in score Mean (SE)	% change	p	Effect size*
**All domains (N = 134)**	2.991 (0.32)	3.113 (0.35)	0.122 (0.030)	4.1	<0.001	0.36
**Perceived risk of heart attack or stroke**	2.551 (0.54)	2.535 (0.60)	-0.016 (0.043)	-0.7	0.702	NA
**Perceived benefits of and intention to exercise**	3.228 (0.46)	3.448 (0.46)	0.220 (0.038)	6.8	<0.001	0.48
**Perceived benefits of and intention to healthy eating**	3.183 (0.57)	3.340 (0.59)	0.157 (0.042)	4.9	<0.001	0.27
**Perceived benefits of and intention to stop smoking (N = 38)**	3.390 (0.81)	3.578 (0.60)	0.187 (0.106)	5.5	0.085	

*—Cohen’s d

There was a significant increase in mean score across all domains of the ABCD questionnaire from 2.99 to 3.11 (p < 0.001). There were also increases in mean score for 3 of the sub-domains of the questionnaire of around 5–7%—these increases in scores were significant for the questions relating to exercise and healthy eating, but did not reach significance for the smoking questions, presumably due to the smaller sample size (n = 38) as these questions were only answered by current smokers. There was no change in the mean score for perceived risk of heart attack or stroke.

Table 8 shows the results of the multiple regression model with age, sex, time spent interacting with the website and initial score as predictors of change in the mean score across all domains of the ABCD questionnaire. Change in score was inversely associated with age and initial score, and increased with increasing time spent interacting with the website. Change in score was not associated with sex in the adjusted model.

**Table 8:** Results of regression model to predict to change in mean score across all domains of the ABCD questionnaire.

	B (SE)	p
**Age (y)**	-0.008 (0.003)	0.003
**Sex = male** ^**a**^	0.040 (0.056)	0.469
**Time spent on website > 20 minutes** ^**b**^	0.115 (0.052)	0.029
**Initial score**	-0.443 (0.082)	<0.001
**Model Summary: Adj R**^**2**^ **= 0.259, F**_**4,129**_ **= 12.645, p < 0.001**		

a–reference category is female b–reference category is under 20 minutes

**Usability:** The mean(sd) score on the SUS survey was 77.5 (13.5) with a range of 30–100, with this being considered a good or B+ rating [33]. 89% of respondents either agreed or strongly agreed that the website was easy to use and 91% that the average person would find it easy to learn.

## Discussion

This paper describes the development and evaluation of www.healthyheartsproject.co.uk. This study found that using the Healthy Hearts Website was associated with an increase in user’s total ABCD scores, and in the sub-components relating to attitudes towards exercise and diet. Change in score was greater for those who spent over 20 minutes engaging with the website. The website was rated as having good usability on the System Usability Scale. In the Thinking Aloud study, participants were generally clear about the purpose of the website and how to use it and the meaning of the graphics used to communicate risk. The questionnaires and feedback were generally felt to be understandable and inclusive of a range of lifestyles and cultures, although with a limited suitability for those on special diets. The main issues raised were with the amount of text and the readability, and the need for users to have a high level of self-motivation to engage with all sections of the website. We have developed and evaluated the website, but further in-practice evaluation and testing of the website as a tool for CHWs still needs to be conducted.

Our findings highlight the importance of simple, concise text and clear, non-judgemental feedback. This is in line with the results of other studies and design recommendations for eHealth products [34,35]. Participants commented on the level of motivation needed to complete all sections of the website, however, they also found the process of completing the questionnaires, in particular on diet, acted in itself as a prompt to re-evaluate their health behaviours. Similar findings have been reported in several other studies of eHealth products focussed around diet and physical activity, where users have found the process of completing the assessments both onerous and illuminating [36,37]. Users valued the personalized feedback they received. Personalized content has been found to be a facilitator of e-Health use by older adults [38], and a meta-analysis of web-delivered tailored behaviour change interventions found positive effects on health outcomes both post-testing and at follow-up [39].

Personal data security was mentioned by the majority of participants, and is a key concern with eHealth products. There have been a number of high profile reports of health apps either sharing or leaking personal data [40,41]. One recent study found code presenting potential privacy concerns in 88% of mHealth apps [42]. Concerns over privacy and security have been found to be a barrier to the use of e-health by older adults [38], and linked to reluctance to download contract-tracing apps for COVID-19[43]. The Thinking Aloud study also revealed potential issues relating to trust in the website, including wishing to see use statistics and more information on the design team, and doubting some of the feedback and advice received—similar concerns over trustworthiness of e-health products have been reported in a number of other studies [36,38,44].

The suitability of the question on sex on the Interheart survey for those who identify as trans and non-binary was raised by several participants in the Thinking Aloud study, and this issue was also discussed by members of the research team and the William Joseph team during the website development process. Data from the UK 2021 census suggests the size of the trans population of England and Wales is around 260,000[45], and it is likely that this number is rising, with referrals to the Gender Identity Development Service rising steeply from 77 in 2009–10 to 2590 in 2018–19[46]. The calculation of CVD risk from the Interheart questionnaire is based on biological sex–good estimates of long-term CVD risk in trans men and women are not yet available and will be affected by issues such as receipt of gender-affirming hormone therapy [47]. However, further consideration needs to be given as to how to best make this question and the calculation of CVD risk inclusive of trans and non-binary individuals.

### Strengths and limitations of study design

Nearly all respondents in Thinking Aloud study were highly educated, with 60% educated to postgraduate level. While this is typical of the CHW volunteers participating in the UK SPICES programme, who were generally well-educated and health literate [48] it is not typical of the materially-deprived target population for the SPICES programme. However, the Thinking Aloud participants were drawn largely from a group of health professionals and volunteers with considerable experience of working with materially deprived communities, and their comments on the website were made with consideration of the issues affecting such communities. The participants in the quantitative survey were more representative of the target population as it was possible to select for those educated to less than university degree level. We also repeated our analysis of the changes in scores from the ABCD questionnaire after completing the website, excluding the most educated participants (the 45% of the sample educated to A level/High school diploma level). The results were very similar–the regression coefficients for age and initial score were almost unchanged (B(se) of -0.008(0.003) and -0.493(0.099) respectively, and the association with time spent on the website was slightly strengthened with the B(se) increasing to 0.168(0.067). It is possible that participants recruited via Prolific may be more digitally literate than both the general population, and the target audience for the website, which may introduce a bias into the results. More women than men took part–this is typical of e-health studies but also reflects the sex-balance of those registered on Prolific (about twice as many women as men). We found significant changes in attitudes to exercise and diet immediately after using the website, and psychological beliefs about the importance of lifestyle to CVD risk have been shown to be associated with the likelihood of lifestyle behaviour change [49]. However, we were unable to conduct a long-term follow-up and so could not assess whether any changes in attitudes and beliefs to CVD were sustained, or to measure any changes in behaviours that might result from using the website or how long these might last.

A further limitation is the representativeness of our sample in terms of ethnicity and age. The sample for the “Thinking Aloud” evaluation was 80% white, information on ethnicity was not available for the Prolific sample but over 90% of respondents were born in the UK. In addition, 60% of the sample for the “Thinking Aloud” evaluation were aged under 55y and around 80% of the Prolific sample were aged under 50y. As a result we have limited data on the suitability of this website for users of colour and from diverse backgrounds, and for older adults, the group at the highest risk of CVD. Younger adults may be more likely to interact with web-based tools. However, although the absolute risk of CVD is lower at younger ages, there is also a greater potential for lifestyle changes to reduce lifetime CVD risk the earlier they are adopted. This is particularly true among materially deprived communities, where the burden of ill health is relatively high at younger ages, with only 39.7% of individuals living in areas of the UK in the highest decile of deprivation reporting being in very good health at age 35-39y, compared with 59.3% in the least deprived decile [50].

### Strengths and limitations of the Healthy Hearts project website

The Healthy Hearts Project website has a number of linked strengths and shortcomings. It allows risk assessment and goal setting for the four key behavioural determinants of CVD risk; smoking, diet, alcohol consumption and physical activity, while most e-health tools concentrate on only one or two target behaviours [51,52]. It delivers detailed personalized feedback based on the user’s input. However, this requires extensive information to be entered by the user and requires a level of time and self-motivation. It does not store personal data, so does not present a privacy risk. The results can be emailed and stored by the person using it. Conversely, the decision not to store data means all sections of the website must be completed in one sitting and it is not directly possible for users to track lifestyle changes over time, although users can look at the previous emails. The decision to exclude hyperlinks to avoid users accidentally losing their progress through the website has meant that it was not possible to include links to external sources of support and information. Given the key role of support to achieving lifestyle change, additional ways to signpost to external sources of support from the website should be investigated. Yardley et al. recommend that digital health-related behaviour change interventions are designed around the perspective and context of the population who will use it, based on repeated development-evaluation-development cycles of qualitative research with the intended users [34]. Intended user perspectives on CHD and behaviour change were taken into account when planning the design of the website, feedback from the Thinking Aloud assessment of the prototype website was incorporated, and further adjustments are planned based on the results of this study.

The website has been designed and tested in the UK and assessed using a predominantly white, UK-born sample. The questionnaires used to assess CVD risk (Interheart) and physical activity levels (IPAQ) have both been internationally validated [29,53], and so these aspects of the website should still have good applicability for users from other countries. However, the dietary questionnaire used has been designed to assess typical British diets–the need for culture-specific food frequency questionnaires is well-recognised [54] and so in its current form website may have limited utility to assess dietary intakes of users from other countries, or from minority ethnic groups within the UK. Some adjustment to the dietary questionnaire used would probably be needed to make the website suitable for use with these groups.

A further limitation of the website is the focus on medical risk. There are a number of ethical issues with such an approach to CVD prevention, particularly in materially deprived communities. These include the individualization of what might better be considered as collective social problems, potential stigmatization of those with “unhealthy” lifestyle behaviours, and possible distress resulting from being assessed as high risk for those with limited resources to address this [55]. Participants in the Thinking Aloud study alluded to this, noting the need to avoid feedback that might feel judgemental, and the community issues that might act as barriers to lifestyle change.

### Use of the Healthy Hearts Project website in the community

For the Healthy Hearts Project website to be an effective lifestyle modification tool, strategies are needed to promote its use and maintain engagement among the target user group. Promotion via NHS primary healthcare practices is one way to achieve this. The Healthy Hearts Project website was used as part of a follow-up to the main SPICES programme known as Healthy Hearts Sussex (HHS), where the CHW-delivered motivational lifestyle coaching programme was delivered via a primary care practice based in a materially deprived community in Sussex rather than via VCSEs. By placing the management of HHS in a primary care practice it was hoped that the reach and sustainability of the intervention could be increased. In addition to the coaching intervention, HHS made use of the Healthy Hearts project website and monitored its use by community members. Users who were not able to take part in the coaching intervention but were interested in improving their CVD health were prompted to use the website as a self-management tool. The Healthy Hearts Project website was shared through the website of the primary care practice—there were 356 unique visitors between April and May 2022 and 51 of these users returned to the website more than once. Primary care practice staff members provided positive feedback about the website, stating that it was well-designed, easy to use, and non-threatening to participants. The data from this study cannot be published as it was produced as an internal NHS report however it shows how the website has been incorporated into a local CVD-prevention pilot project as a self-management option.

## Conclusions and general lessons learned

The Healthy Hearts Project website was developed in a user-led design, was generally rated as usable by both quantitative and qualitative measures and resulted in a change in CVD attitudes/knowledge—an important precursor to lifestyle change. How it would best support community led health coaching programmes would need to be explored further. There is potential for numerous other roles, including those who sit in primary care settings (nurses, health coaches, social prescribers etc.) and those working in the wider community (e.g in VCSEs) to introduce people to the website or similar mHealth tools. A number of lessons can be drawn from the results of this study, these are:

1. Text used should be simple and concise throughout
2. Feedback should be clear, non-judgemental and personalized.
3. It is important to create trust, both in the website’s credentials and in the security of any personal data
4. Detailed lifestyle assessments can act as a prompt for health behaviour evaluation, but strategies to maintain motivation to complete all stages of any assessments should be considered.

These lessons can be used to improve future versions of the website and have general relevance to the design of similar eHealth products.

## Supporting information

S1 Fig Example of the website’s colour scheme. (TIF)

S1 Appendix Design Phase Thinking Aloud Discussion Guide 1. (DOCX)

S2 Appendix Design Phase Thinking Aloud Discussion Guide 2. (DOCX)

S3 Appendix Evaluation Phase Thinking Aloud Discussion Guide. (DOCX)

S4 Appendix System Usability Scale. (DOCX)

S5 Appendix Interheart Questionnaire with scoring. (DOCX)

S6 Appendix International Physical Activity Questionnaire with scoring. (DOCX)
