# Supplementary material for: The Healthy Hearts Project: Development and evaluation of a website for cardiovascular risk assessment and visualisation and self-management through healthy lifestyle goal-setting
Source: PLOS Digit Health. 2023 Nov 29;2(11):e0000395. doi: 10.1371/journal.pdig.0000395 (PMC10686463; doi:10.1371/journal.pdig.0000395)
Supplement: S1 Appendix — (DOCX) [file pdig.0000395.s002.docx]

**Appendix 1. Design Phase Thinking Aloud Discussion Guide 1**

**Testing Script - User journey 1_0**

**Ahead of the calls:**

We will be asking you to share your screen with us.

We will talk everyone through this process, or you can find out more on these links here:

<https://support.zoom.us/hc/en-us/articles/201362153-Sharing-your-screen>

# Start test

Hi, **<*Tester>***. My name is James, and I’m going to be taking you through this session today.

- I work for William Joseph working with the team from Brighton & Sussex Medical School
- We’re creating a website that helps people understand their heart health and set goals for improving it
- Today we’re looking at a series of pages on the website to find out if they work as you expect them to. It’s still a prototype
- We’re testing the pages, not you
- You can’t do anything wrong so don’t worry about making mistakes
- Be honest with your opinions
- Think out loud
  - say what you’re looking at
  - say what you’re trying to do
  - say what you’re thinking
  - say if anything is not as you expect
- Any questions as we go, just ask - I might not give you the answer right away as I’m going to let you figure things out for yourself without prompting you too much.
- We are going to be recording session today - this is just for the project team and won’t be shared more widely
- Please confirm you’re ok for us to record the session
- If you need to take a break that’s fine, just let me know
- We’ve got some observers watching who will be taking notes ● **<intro observers>**

# Contextual questions

1. What do you know about Cardiovascular disease?
2. Have you ever spoken to a medical professional about your Cardiovascular disease risk?
3. Has anyone in your family been diagnosed with heart disease?
4. How do you know the Brighton & Sussex Medical School?
5. Do you take any specific steps to minimise your risk of heart disease?
6. Do you smoke? Do you drink?
7. How often do you exercise and what type?
8. How often do you eat your 5 a day for fruit and vegetables?
9. Do you use a fitbit, apple watch or other technology to help monitor your health?
10. What are your main worries in life at the moment?
11. Have you participated in an online research project before?
12. Have you used online services with your doctor’s surgery or similar?
13. Can you describe what the average day is like for you in terms of your use of technology - ie what devices do you use and what do you use them for?

**Homepage - intro and start of test:**

[**https://www.figma.com/proto/Z3Axqb63HWA1MIzAtPs sXC/WJ4021-SPICES-Prototype-3_0?node-id=1%3A546 &scaling=min-zoom**](https://www.figma.com/proto/Z3Axqb63HWA1MIzAtPssXC/WJ4021-SPICES-Prototype-3_0?node-id=1%3A546&scaling=min-zoom)

**Initial response** *(after 5 seconds looking over the page)*

1. What is this website about?
2. Who is it by?
3. How would you expect it to help you?
4. How did it make you feel?

SUCCESS: *Sussex University, Heart health, information, trustworthy*

## Now take a longer look

1. Can you say a bit more about what it’s about?
2. Do you know what the process will entail?
3. Where would you click to proceed?

SUCCESS:

*Any of the project aims*

*Know they will require a tape measure*

*Identify the navigation*

**Interheart questionnaire:**

[**https://www.figma.com/proto/Z3Axqb63HWA1MIzAtPs sXC/WJ4021-SPICES-Prototype-3_0?node-id=1%3A650 &scaling=min-zoom**](https://www.figma.com/proto/Z3Axqb63HWA1MIzAtPssXC/WJ4021-SPICES-Prototype-3_0?node-id=1%3A650&scaling=min-zoom)

**Initial response** *(show for 5 seconds and then ask participant what they see)*

1. What is this page about?
2. How many questions will you be asked?
3. Where would you click to proceed?

SUCCESS: *identify key information types on page*

## Now take a longer look

1. Is there any information you think is missing on the page?
2. Where would you click if you wanted to correct a previous question?

## Click through the initial questions

1. How do you feel about the amount of questions on each page? Show me how you would answer each of them
2. How do you feel about the wording/nature of the questions?
3. Would you want the option to return at a later stage?

**Physical factors (page 4 of Interheart questionnaire):** [**https://www.figma.com/proto/Z3Axqb63HWA1MIzAtPs sXC/WJ4021-SPICES-Prototype-3_0?node-id=78%3A11 8&scaling=min-zoom**](https://www.figma.com/proto/Z3Axqb63HWA1MIzAtPssXC/WJ4021-SPICES-Prototype-3_0?node-id=78%3A118&scaling=min-zoom)

1. What do you need to do on this page?

SUCCESS: *reflective description*

1. Is there anything that might make it clearer?

**Interheart results page:**

[**https://www.figma.com/proto/Z3Axqb63HWA1MIzAtPs sXC/WJ4021-SPICES-Prototype-3_0?node-id=1%3A781 &scaling=min-zoom**](https://www.figma.com/proto/Z3Axqb63HWA1MIzAtPssXC/WJ4021-SPICES-Prototype-3_0?node-id=1%3A781&scaling=min-zoom)

**5 second test** *(show for 5 seconds and then take away, asking participant what they remember)*

1. What is this page about?
2. How would you expect it to help you?

SUCCESS: *reflective explanation*

## Now take a longer look

1. What risk group are you shown to be in?

SUCCESS: *Green*

1. What does that mean in terms of your heart health?

SUCCESS: *No higher risk*

1. What more would you want to know?
2. Where would you go for smoking information?

SUCCESS: *Smoking: find out more*

1. Where would you go to receive your results via email?

SUCCESS: *Identify full breakdown bar and email input*

1. Where would you go to learn more?

SUCCESS: *Find out more link at the bottom of page*

1. What do you expect to find when you click through?

**Questionnaire dashboard:**

[**https://www.figma.com/proto/Z3Axqb63HWA1MIzAtPs sXC/WJ4021-SPICES-Prototype-3_0?node-id=128%3A7**](https://www.figma.com/proto/Z3Axqb63HWA1MIzAtPssXC/WJ4021-SPICES-Prototype-3_0?node-id=128%3A75&scaling=min-zoom)

[**5&scaling=min-zoom**](https://www.figma.com/proto/Z3Axqb63HWA1MIzAtPssXC/WJ4021-SPICES-Prototype-3_0?node-id=128%3A75&scaling=min-zoom)

1. What is this page about?

SUCCESS: *Further questionnaires and feedback*

1. How long will the other questionnaires take to complete?

SUCCESS: *10 minutes*

1. What do you expect to find when you click through?

**DDQ Diet:**

[**https://www.figma.com/proto/Z3Axqb63HWA1MIzAtPs sXC/WJ4021-SPICES-Prototype-3_0?node-id=11%3A128 &scaling=min-zoom**](https://www.figma.com/proto/Z3Axqb63HWA1MIzAtPssXC/WJ4021-SPICES-Prototype-3_0?node-id=11%3A128&scaling=min-zoom)

1. How long is the questionnaire asking you to think back?

SUCCESS: *1 month*

1. How many questions are there in this questionnaire?

SUCCESS: *21*

1. With this in mind, how does answering one question at a time make you feel?

**DDQ results:**

[**https://www.figma.com/proto/Z3Axqb63HWA1MIzAtPs sXC/WJ4021-SPICES-Prototype-3_0?node-id=128%3A1 416&scaling=min-zoom**](https://www.figma.com/proto/Z3Axqb63HWA1MIzAtPssXC/WJ4021-SPICES-Prototype-3_0?node-id=128%3A1416&scaling=min-zoom)

1. Where would you click to get a more detailed breakdown of your results?

SUCCESS: *Read a more detailed breakdown*

**DDQ breakdown:**

[**https://www.figma.com/proto/Z3Axqb63HWA1MIzAtPs sXC/WJ4021-SPICES-Prototype-3_0?node-id=128%3A6 80&scaling=min-zoom**](https://www.figma.com/proto/Z3Axqb63HWA1MIzAtPssXC/WJ4021-SPICES-Prototype-3_0?node-id=128%3A680&scaling=min-zoom)

1. Where would you look to read more about your fruit intake?

SUCCESS: *Identifies fruit and vegetables – either from sub-menu or by scrolling*

*(pass)*

1. Where would you look to set yourself goals to improve?

SUCCESS: *Set diet goals*

**DDQ goal setting:**

[**https://www.figma.com/proto/Z3Axqb63HWA1MIzAtPs sXC/WJ4021-SPICES-Prototype-3_0?node-id=128%3A1 028&scaling=min-zoom**](https://www.figma.com/proto/Z3Axqb63HWA1MIzAtPssXC/WJ4021-SPICES-Prototype-3_0?node-id=128%3A1028&scaling=min-zoom)

1. Which options did the user select previously?

SUCCESS: *Identify pre-selected option*

1. Which options would give a better score?

SUCCESS: *Identify green options*

1. Which options would give a worse score?

SUCCESS: *Identify red options*

**DDQ goal setting results:**

[**https://www.figma.com/proto/Z3Axqb63HWA1MIzAtPs sXC/WJ4021-SPICES-Prototype-3_0?node-id=128%3A1 416&scaling=min-zoom**](https://www.figma.com/proto/Z3Axqb63HWA1MIzAtPssXC/WJ4021-SPICES-Prototype-3_0?node-id=128%3A1416&scaling=min-zoom)

1. Where would you go to get results emailed to you?

*SUCCESS: Identify email banner*

1. What would you expect to receive in the email at this stage?

*SUCCESS: Interheart + diet – Results and diet goals*
