# Supplementary material for: The Healthy Hearts Project: Development and evaluation of a website for cardiovascular risk assessment and visualisation and self-management through healthy lifestyle goal-setting
Source: PLOS Digit Health. 2023 Nov 29;2(11):e0000395. doi: 10.1371/journal.pdig.0000395 (PMC10686463; doi:10.1371/journal.pdig.0000395)
Supplement: S2 Appendix — (DOCX) [file pdig.0000395.s003.docx]

**Appendix 2. Design Phase Thinking Aloud Discussion Guide 2**

**Testing Script - User journey full design 1_0**

**Ahead of the calls:**

We will be asking you to share your screen with us.

We will talk everyone through this process, or you can find out more on these links here:

<https://support.zoom.us/hc/en-us/articles/201362153-Sharing-your-screen>

# Add schedule

**Testing will focus on the following screens:**

Current FIGMA link

[https://www.figma.com/proto/zFrYMql6aWtQVWpxAoWaM0/WJ4021-SPICES-Design-6_0?nod e-id=1208%3A7840&viewport=235%2C243%2C0.07199130952358246&scaling=min-zoom&hi de-ui=1](https://www.figma.com/proto/zFrYMql6aWtQVWpxAoWaM0/WJ4021-SPICES-Design-6_0?node-id=1208%3A7840&viewport=235%2C243%2C0.07199130952358246&scaling=min-zoom&hide-ui=1)

1. Interheart landing
2. Interheart questions (all)
3. Interheart results
4. Detailed results
5. Diet questionnaire landing
6. Skip to Diet questionnaire results
7. Diet goals landing
8. Diet goal setting
9. Diet goals results
10. Exercise - days/hours pattern (if time)

# Start test

Hi, **<*Tester>***. My name is Nic, and I’m going to be taking you through this session today.

- I work for William Joseph working with the team from Brighton & Sussex Medical School
- We’re creating a website that helps people understand their heart health and set goals for improving it
- We’ve built a model of the website to test with you…
- We’re testing the pages, not you
- You can’t do anything wrong so don’t worry about making mistakes
- Be honest with your opinions
- Think out loud
  - say what you’re looking at
  - say what you’re trying to do
  - say what you’re thinking
  - say if anything is not as you expect
- Any questions as we go, just ask - I might not give you the answer right away as I’m going to let you figure things out for yourself without prompting you too much.
- We are going to be recording session today - this is just for the project team and won’t be shared more widely
- Please confirm you’re ok for us to record the session
- If you need to take a break that’s fine, just let me know
- We’ve got some observers watching who will be taking notes ● **<intro observers>**

# Contextual questions/warm up

**Coach/Connected to BSMS/Spices - GREEN**

**Individuals - GREEN AND BLUE**

- How do you know the Brighton & Sussex Medical School?
- Describe an average day ref. use of technology - what devices do you use and what for?
- Do you use a fitbit, apple watch or other technology to help monitor your health?
- Have you used online services with your doctor’s surgery or similar?
- Prompt example of a good/bad experience online?
- Have you participated in an online research project before?
- What do you know about heart disease?
- Have you ever spoken to a medical professional about your heart disease risk?

## Interheart questionnaire landing page

***5 second test -*** *show for 5 seconds. Then ask participant to minimise their browser and tell us what they remember (it’s not a memory test, just tell us what you noticed).*

[https://www.figma.com/proto/zFrYMql6aWtQVWpxAoWaM0/WJ4021-SPICES-Design-6_0?nod e-id=1208%3A7840&viewport=235%2C243%2C0.07199130952358246&scaling=min-zoom&hi de-ui=1](https://www.figma.com/proto/zFrYMql6aWtQVWpxAoWaM0/WJ4021-SPICES-Design-6_0?node-id=1208%3A7840&viewport=235%2C243%2C0.07199130952358246&scaling=min-zoom&hide-ui=1)

1. What is this page about?

*It’s a questionnaire to understand risk of heart disease*

1. How would you expect it to help you? *helps me understand my risk and make changes to my lifestyle to improve my health*
2. How did it make you feel?

*Trustworthy*

### Now take a longer look

4. How many questions will you be asked?

- 7 questions

5. Does the page tell you about anything you need to do before you start?

- Mentions tape measure

6. Where would you click to proceed?

- Clicks Get started to move to Q1 7. What do you expect will happen next?
- Go to question 1

8. Is there any information you think is missing on this page?

## Interheart questionnaire: Question pages

[https://www.figma.com/proto/zFrYMql6aWtQVWpxAoWaM0/WJ4021-SPICES-Design-6_0?nod e-id=1208%3A7925&viewport=235%2C243%2C0.07199130952358246&scaling=min-zoom&hi de-ui=1](https://www.figma.com/proto/zFrYMql6aWtQVWpxAoWaM0/WJ4021-SPICES-Design-6_0?node-id=1208%3A7925&viewport=235%2C243%2C0.07199130952358246&scaling=min-zoom&hide-ui=1)

### For Q1 – About you

9. What do you have to do on this page?

- understand the questions they are being asked

1. Is there any information you think is missing on this page?
2. Does the page tell you how many questions you have left?

- Can find progress bar and understand where they are in the process 12. What do you think will happen next?
- use ‘Next’ to move to next question

13. What would you do if you wanted to go back a step?

- Can use ‘Back’ to check/change previous answer

For Q2 – Smoking

14. Show me what you would do on this page

- understand the questions they are being asked

For Q3 – Medical history

15. Show me what you would do on this page

- understand the questions they are being asked

For Q4 – Physical factors

16. Show me what you would do on this page

- understand the questions they are being asked

17. Does the page tell you how to measure your waist/hips?

- understand how to measure

For Q5 – Additional factors

18. Show me what you would do on this page

- understand the questions they are being asked

For Q6 – Dietary factors

19. Show me what you would do on this page

- understand the questions they are being asked

For Q7 – Physical activity

20.Show me what you would do on this page What do you expect to happen next?

**Interheart results page:**

[https://www.figma.com/proto/zFrYMql6aWtQVWpxAoWaM0/WJ4021-SPICES-Design-6_0?nod e-id=1208%3A8082&viewport=235%2C243%2C0.07199130952358246&scaling=min-zoom&hi de-ui=1](https://www.figma.com/proto/zFrYMql6aWtQVWpxAoWaM0/WJ4021-SPICES-Design-6_0?node-id=1208%3A8082&viewport=235%2C243%2C0.07199130952358246&scaling=min-zoom&hide-ui=1)

***5 second test -*** *show for 5 seconds. Then ask participant to minimise their browser and tell us what they remember (it’s not a memory test, just tell us what you noticed).*

21. What is this page about? results page based on questions answered

22.How would you expect it to help you? Mentions risk level

23.How did it make you feel?

- trustworthy, reassuring

### Now take a longer look

24.Tell me about this page, what can you do?

- Can find onward journeys to detailed results, next questionnaires, smoking goals and email results

25.What risk group are you in? Does the page tell you what that risk means?

> Prompt risk range bar

- Understands risk level

26. What is the page asking you to do next?

- Detailed results

27. Show me how you would find out more information based on your results?

- Can proceed to detailed results

28. What do you expect will happen next…

29.Is there any information you think is missing on this page?

> Prompt ‘Now go to Detailed results’

**-> IF SUCCESSFUL GO TO DETAILED RESULTS BREAKDOWN Q41**

30.Where would you go to get your results by email?

- Can find email results section

31. What do you expect will happen next?

32.Does the page give you any other sources of information you can trust?

33.Can you show me where you are in the process?

> Prompt ‘Progress’ menu

- Knows where they are in the process 34.Does the page tell you what to do next?

> Prompt Get started (diet or exercise)

- Can proceed to next questionnaire

35. What do you expect will happen next…

36.Is there any information you think is missing on this page?

## Detailed results breakdown

[https://www.figma.com/proto/zFrYMql6aWtQVWpxAoWaM0/WJ4021-SPICES-Design-6_0?nod e-id=1208%3A12494&viewport=235%2C243%2C0.07199130952358246&scaling=min-zoom& hide-ui=1](https://www.figma.com/proto/zFrYMql6aWtQVWpxAoWaM0/WJ4021-SPICES-Design-6_0?node-id=1208%3A12494&viewport=235%2C243%2C0.07199130952358246&scaling=min-zoom&hide-ui=1)

***5 second test -*** *show for 5 seconds. Then ask participant to minimise their browser and tell us what they remember (it’s not a memory test, just tell us what you noticed).*

37. What is this page about? Detailed results page

38.How would you expect it to help you?

Get personalised advice about different areas of heart health

39.How did it make you feel?

- trustworthy, reassuring, informative, relevant

### Now take a longer look

40.Tell me about this page, what can you do?

- Understands information

41. Show me how you would find out more information about smoking?

- Can show/hide information sections

42.Does the page give you any other sources of information you can trust?

- Mentions other trusted sources of information

43.Is there any information you think is missing on this page?

44. Where would you go to get your results by email?

- Can find email results section

45. Now show me how you would get to the next section

- Can go back to Results page

**<- THEN GO BACK TO INTERHEART RESULTS PAGE Q26**

## Diet questionnaire landing page

[https://www.figma.com/proto/zFrYMql6aWtQVWpxAoWaM0/WJ4021-SPICES-Design-6_0?nod e-id=1208%3A7862&viewport=235%2C243%2C0.07199130952358246&scaling=min-zoom&hi de-ui=1](https://www.figma.com/proto/zFrYMql6aWtQVWpxAoWaM0/WJ4021-SPICES-Design-6_0?node-id=1208%3A7862&viewport=235%2C243%2C0.07199130952358246&scaling=min-zoom&hide-ui=1)

46.What is this page about?

- Understands information

47. How many questions will you be asked?

- 21 questions

48.How long will you need to complete this questionnaire?

- 20 minutes

49.Where would you click to proceed?

- Get started

50.What do you expect will happen next?

- Go to Q1

51. Is there any information you think is missing on this page?

## Diet questionnaire Question 1 - Vegetables

52.Show me what you would do on this page

- understand the questions they are being asked

## Now we will click to results - imagine you’ve completed the questionnaire

**Diet results page:**

[https://www.figma.com/proto/zFrYMql6aWtQVWpxAoWaM0/WJ4021-SPICES-Design-6_0?nod e-id=1208%3A8105&viewport=235%2C243%2C0.07199130952358246&scaling=min-zoom&hi de-ui=1](https://www.figma.com/proto/zFrYMql6aWtQVWpxAoWaM0/WJ4021-SPICES-Design-6_0?node-id=1208%3A8105&viewport=235%2C243%2C0.07199130952358246&scaling=min-zoom&hide-ui=1)

53.Tell me about this page, what can you do?

- Can find onward journeys to detailed results, goal setting, next questionnaire, and email results

54.What risk group are you in? Does the page tell you what that risk means?

> Prompt risk range bar

- Understands risk range

55. Imagine you have reviewed your ‘detailed results’. What is the page asking you to do next?

- Setting diet goals
- Completing exercise questionnaire
- Setting smoking goals

1. What do you expect will happen next…
2. Is there any information you think is missing on this page?

> Prompt ‘Set your diet goals’

**Diet goals landing page:**

[https://www.figma.com/proto/zFrYMql6aWtQVWpxAoWaM0/WJ4021-SPICES-Design-6_0?nod e-id=1208%3A7904&viewport=235%2C243%2C0.07199130952358246&scaling=min-zoom&hi de-ui=1](https://www.figma.com/proto/zFrYMql6aWtQVWpxAoWaM0/WJ4021-SPICES-Design-6_0?node-id=1208%3A7904&viewport=235%2C243%2C0.07199130952358246&scaling=min-zoom&hide-ui=1)

58.What is this page about?

- Understand it’s start page for setting diet goals 59.How many questions will you be asked?
- 10 questions

60.How long will you need to set your diet goals?

- 10 minutes

61. Where would you click to proceed?

- Get started

62.What do you expect will happen next?

63.Is there any information you think is missing on this page?

**Diet goal setting page (vegetables):**

[https://www.figma.com/proto/zFrYMql6aWtQVWpxAoWaM0/WJ4021-SPICES-Design-6_0?nod e-id=1208%3A7789&viewport=235%2C243%2C0.07199130952358246&scaling=min-zoom&hi de-ui=1](https://www.figma.com/proto/zFrYMql6aWtQVWpxAoWaM0/WJ4021-SPICES-Design-6_0?node-id=1208%3A7789&viewport=235%2C243%2C0.07199130952358246&scaling=min-zoom&hide-ui=1)

64.What is this page about

- Understands its about setting goals

65.What are being asked to do? Prompt: Why do you think that option is highlighted? [could you eat a portion of vegetables more often?]

- Understands highlighted option relates to their own result, understands the page is asking them to select an option for improvement

66.Show me what you would do next to set your [vegetable diet goal]

- Understand free text box for adding your own goal

67. Does the page give you any other sources of information you can trust?

- Can find trusted information sources 68. What is the page asking you to do next?
- Can proceed to next page

1. What do you expect will happen next…
2. Is there any information you think is missing on this page?

Imagine you’ve completed all your goal setting pages

**Diet goals results page:**

[https://www.figma.com/proto/zFrYMql6aWtQVWpxAoWaM0/WJ4021-SPICES-Design-6_0?nod e-id=1208%3A8126&viewport=235%2C243%2C0.07199130952358246&scaling=min-zoom&hi de-ui=1](https://www.figma.com/proto/zFrYMql6aWtQVWpxAoWaM0/WJ4021-SPICES-Design-6_0?node-id=1208%3A8126&viewport=235%2C243%2C0.07199130952358246&scaling=min-zoom&hide-ui=1)

1. What is this page about?

- Understand goals summary

72. What is it telling you about your risk group?

> Prompt risk range bar

- Understand how risk range is showing improvement 73. Where would you go to get your results by email?
- Can find email results section

1. Does the page give you any other sources of information you can trust?
2. Can you show me where you are in the process?

> Prompt ‘Progress’ menu

Tell me what the progress menu is showing you?

- Can find progress section and knows where they are in process 76. What is the page asking you to do next?
- Go to exercise questionnaire

77. Is there any information you think is missing on this page?

**Exercise questionnaire:**

[https://www.figma.com/proto/zFrYMql6aWtQVWpxAoWaM0/WJ4021-SPICES-Design-6_0?nod e-id=1208%3A7883&viewport=235%2C243%2C0.07199130952358246&scaling=min-zoom&hi de-ui=1](https://www.figma.com/proto/zFrYMql6aWtQVWpxAoWaM0/WJ4021-SPICES-Design-6_0?node-id=1208%3A7883&viewport=235%2C243%2C0.07199130952358246&scaling=min-zoom&hide-ui=1)

## Wrap up

- How did you feel using the website?
- Is there anything else you’d like to share about your experience today?

### Thank you, stop recording, end interview
