# Supplementary material for: The Healthy Hearts Project: Development and evaluation of a website for cardiovascular risk assessment and visualisation and self-management through healthy lifestyle goal-setting
Source: PLOS Digit Health. 2023 Nov 29;2(11):e0000395. doi: 10.1371/journal.pdig.0000395 (PMC10686463; doi:10.1371/journal.pdig.0000395)
Supplement: S3 Appendix — (DOCX) [file pdig.0000395.s004.docx]

**Appendix 3. Evaluation Phase Thinking Aloud Discussion Guide**

| **Completed website qualitative evaluation**  **Thinking aloud – discussion guide** | | |
| --- | --- | --- |
| We will be asking you to share your screen with us.  We will talk everyone through this process, or you can find out more on these links here:   1. Interheart landing 2. Interheart questions (all) 3. Interheart results 4. Detailed results 5. Diet/Exercise questionnaire landing 6. Diet/Exercise question 7. Diet/Exercise questionnaire results 8. Setting goals landing 9. Goal setting 10. Setting goals results   Hi, **<*participant>***. My name is ___, and I’m going to be taking you through this session today.   - I work for Brighton & Sussex Medical School - We’ve created a website that helps people understand their heart health and set goals for improving it - We’re testing the website, not you - You can’t do anything wrong so don’t worry about making mistakes - Be honest with your opinions - Think out loud - say what you’re looking at - say what you’re trying to do - say what you’re thinking - say if anything is not as you expect - Any questions as we go, just ask - I might not give you the answer right away as I’m going to let you figure things out for yourself without prompting you too much. - We are going to be recording session today - this is just for the project team and won’t be shared more widely - Please confirm you’re ok for us to record the session - If you need to take a break that’s fine, just let me know - We’ve got some observers watching who will be taking notes | | |
| BEGIN THINKING ALOUT EXERCISE   - If participant is silent for more than 20 second   - PROMPT – ‘THINK ALOUD’ | | |
| **Contextual questions/warm up**  **Coach/Connected to BSMS/Spices - GREEN**  • How do you know the Brighton & Sussex Medical School?  • Describe an average day ref. use of technology - what devices do you use and what for?  • Do you use a Fitbit, apple watch or other technology to help monitor your health?  • Have you used online services with your doctor’s surgery or similar?  •  • Have you participated in an online research project before?  • What do you know about heart disease?  • Have you ever spoken to a medical professional about your heart disease risk? | | |
| **OPENING SCREEN**  ***< 5 second test -*** *show for 5 seconds. Then ask participant to minimise their browser and tell us what they remember (it’s not a memory test, just tell us what you noticed).* ***>*** | | |
| 1. What is this page about? |  | SUCCESS IF: It’s a questionnaire to understand risk of heart disease |
| 1. How would you expect it to help you? |  | SUCCESS IF: helps me understand my risk and make changes to my lifestyle to improve my health |
| 1. How did it make you feel? |  |  |
| **< Now take a longer look >** | | |
| 1. What do you think this website is about? |  | SUCCESS IF: Understand it’s start page for heart health questionnaire |
| 1. Does the page tell you about anything you need to do before you start? |  | SUCCESS IF: it mentioned tape measure |
| 1. Where would you click to proceed? |  | SUCCESS IF: Clicks Get started to move to Q1 |
| 1. What do you expect will happen next? |  | SUCCESS IF: Go to questionnaire |
| 1. Is there any information you think is missing on this page? |  |  |
| **Heart Health questionnaire landing page**  ***< 5 second test -*** *show for 5 seconds. Then ask participant to minimise their browser and tell us what they remember (it’s not a memory test, just tell us what you noticed).****>*** | | |
| 1. What is this page about? |  | SUCCESS IF: It’s a questionnaire to understand risk of heart disease |
| 1. How would you expect it to help you? |  | SUCCESS IF: helps me understand my risk and make changes to my lifestyle to improve my health |
| 1. How did it make you feel? |  |  |
| **< NOW TAKE A LONGER LOOK >** | | |
| 1. What is this page about? |  | SUCCESS IF: Understand it’s start page for heart health questionnaire |
| 1. Does the page tell you about anything you need to do before you start? |  | SUCCESS IF: Mentions tape measure |
| 1. Where would you click to proceed? |  | SUCCESS IF: Clicks Get started to move to Q1 |
| 1. Is there any information you think is missing on this page? |  |  |
| **Interheart questionnaire: Question pages**  < If participant is silent for more than 20 seconds>  > PROMPT – ‘THINK ALOUD’  <Ask the following questions whilst they are answering the questions > | | |
| 1. What do you have to do on this page? |  | SUCCESS IF: understand the questions they are being asked |
| 1. Is there any information you think is missing on this page? |  |  |
| 1. Do you understand why you are being asked these questions? |  |  |
| 1. Can you tell where you are in the questionnaire? |  | SUCCESS IF: Can find progress bar and understand where they are in the process |
| 1. What do you think will happen next? |  | SUCCESS IF: use ‘Next’ to move to next question |
| 1. What would you do if you wanted to go back a step? |  | SUCCESS IF: Can use ‘Back’ to check/change previous answer |
| 1. Does the page tell you how to measure your waist/hips? |  | SUCCESS IF: Understand how to measure |
| **Interheart results page:**  ***5 second test -*** *show for 5 seconds. Then ask participant to minimise their browser and tell us what they remember (it’s not a memory test, just tell us what you noticed).* | | |
| 1. What is this page about? |  |  |
| 1. How did it make you feel? |  |  |
| **<Now take a longer look>** | | |
| 1. Tell me about this page, what can you do? |  | SUCCESS IF: Can find onward journeys to detailed results, next questionnaires, smoking goals and email results |
| 1. What risk group are you in? Does the page tell you what that risk means? | <Prompt> to check risk level. | SUCCESS IF: Understands risk level |
| 1. Is there any information you think is missing? |  |  |
| 1. What is the page asking you to do next? |  | SUCCESS IF: Search for detailed results  IF SUCCESSFUL GO TO DETAILED RESULTS BREAKDOWN |
| **Detailed results breakdown** | | |
| 1. Tell me about this page, what can you do? |  | SUCCESS IF: Understands information |
| 1. How would you expect it to help you? |  | SUCCESS IF: 33. Get personalised advice about different areas of heart health |
| 1. How would you check your personalised health advice? |  | SUCCESS IF: Can show/hide information sections |
| 1. Does the page give you any other sources of information you can trust? |  | SUCCESS IF: Mentions other trusted sources of information |
| 1. Is there any information you think is missing on this page? |  |  |
| 1. Now show me how you would get to the next section |  | SUCCESS IF: Can go back to Results page  THEN GO TO EMAIL RESULTS SECTION |
| **Email results section** | | |
| 1. Where would you go to get your results by email? |  |  |
| 1. What do you expect will happen next? | > Prompt ‘Progress’ menu | SUCCESS IF: Knows where they are in the process |
| 1. Does the page tell you what to do next? | > Prompt Get started (diet or exercise) | SUCCESS IF: Can proceed to next questionnaire |
| 1. Is there any information you think is missing on this page? |  |  |
| **Diet and exercise questionnaire landing page**  ***5 second test -*** *show for 5 seconds. Then ask participant to minimise their browser and tell us what they remember (it’s not a memory test, just tell us what you noticed).* | | |
| 1. What is this page about? |  | SUCCESS IF: Understands information |
| 1. How did it make you feel? |  |  |
| **<Now take a longer look>** | | |
| 1. How many questions will you be asked? |  | SUCCESS IF: 26 questions |
| 1. How long will you need to complete this questionnaire? |  | SUCCESS IF: 20 minutes |
| 1. Where would you click to proceed? |  | SUCCESS IF: Get started button |
| 1. Is there any information you think is missing on this page? |  |  |
| **Diet questionnaire Questions**  < If participant is silent for more than 20 seconds>  > PROMPT – ‘THINK ALOUD’  <Ask the following questions whilst they are answering the questions > | | |
| 1. Show me what you would do on this page |  | SUCCESS IF: understand the questions they are being asked |
| 1. How do these questions make you feel? |  |  |
| 1. Why do you think you are being asked these questions? |  |  |
| 1. Are the questions easy to understand? |  |  |
| 1. Are the questions easy to answer? |  |  |
| 1. What do you think of the food items we asked about during the diet questionnaire? |  |  |
| 1. Do you understand vigorous / moderate exercise means? |  |  |
| 1. Is there any information you think is missing on this page? |  |  |
| **Diet results page:** | | |
| 1. Tell me about this page, what can you do? |  | SUCCESS IF: Can find onward journeys to detailed results, goal setting, and email results |
| 1. What risk group are you in? Does the page tell you what that risk means? | > Prompt risk range bar | SUCCESS IF: Understands risk range |
| 1. Imagine you have reviewed your ‘detailed results’. What is the page asking you to do next? | > Prompt ‘Set your diet goals’ | SUCCESS IF: Setting diet goals |
| 1. Is there any information you think is missing on this page? |  |  |
| **Setting goals landing page:** | | |
| 1. What is this page about? |  | SUCCESS IF: Understand it’s start page for setting diet goals |
| 1. Where would you click to proceed? |  |  |
| 1. What do you expect will happen next? |  |  |
| 1. Is there any information you think is missing on this page? |  |  |
| **Setting Goals Section**  < If participant is silent for more than 20 seconds>  > PROMPT – ‘THINK ALOUD’  < Ask the following questions whilst they are answering the questions > | | |
| 1. What is this page about |  | SUCCESS IF: Understands it’s about setting goals |
| 1. What are being asked to do? | > Prompt: Why do you think that option is highlighted? [could you eat a portion of vegetables more often?] | SUCCESS IF: Understands highlighted option relates to their own result, understands the page is asking them to select an option for improvement |
| 1. Show me what you would do next to set your goal |  | SUCCESS IF: Understand free text box for adding your own goal |
| 1. Does the page give you useful information? |  | SUCCESS IF: Can find useful information sources |
| **Setting goals results page:** | | |
| 1. What is this page about? |  | SUCCESS IF: Understand goals summary |
| 1. What is it telling you about your risk group? | > Prompt risk range bar | SUCCESS IF: Understand how risk range is showing improvement |
| 1. Where would you go to get your results by email? |  | SUCCESS IF: Can find email results section |
| 1. Does the page give you any other sources of information you can trust? |  | SUCCESS IF: Can Find information |
| 1. Can you request an email using the tester in email address? | > Enter tester email address | SUCCESS IF: Can Enter Tester Email address |
| **Personalised email result:**  ***5 second test -*** *show for 5 seconds. Then ask participant to minimise their browser and tell us what they remember (it’s not a memory test, just tell us what you noticed).* | | |
| 1. What is this email about? |  | SUCCESS IF: Understands information |
| 1. How did it make you feel? |  |  |
| **<Now take a longer look>** | | |
| 1. How does this email make you feel? |  |  |
| 1. How would you use this email? |  |  |
| 1. Is there any information you would like to see added/removed in this email? |  |  |
| **Wrap up**  < Close internet browser and stope sharing screen > | | |
| 1. How did you feel using the website? |  |  |
| 1. Could you see yourself using this tool to help you make healthy lifestyle changes? |  |  |
| 1. Is there anything else you’d like to share about your experience today? |  |  |
| **DEBRIEF**  <Stop recording and thank participant of taking part in the exercise>  <offer the participant a debrief by offering to answer any questions they have> | | |
