# Supplementary material for: The Healthy Hearts Project: Development and evaluation of a website for cardiovascular risk assessment and visualisation and self-management through healthy lifestyle goal-setting
Source: PLOS Digit Health. 2023 Nov 29;2(11):e0000395. doi: 10.1371/journal.pdig.0000395 (PMC10686463; doi:10.1371/journal.pdig.0000395)
Supplement: S4 Appendix — (DOCX) [file pdig.0000395.s005.docx]

**Appendix 4. System Usability Scale**

|  | Strongly disagree | Disagree | Strongly agree | Not applicable | Strongly disagree |
| --- | --- | --- | --- | --- | --- |
|  | 1 | 2 | 3 | 4 | 5 |
| 1. I think that I would like to use this website frequently |  |  |  |  |  |
| 2. I found this website unnecessarily complex |  |  |  |  |  |
| 3. I thought this website was easy to use |  |  |  |  |  |
| 4. I think that I would need assistance to be able to use this website |  |  |  |  |  |
| 5. I found the various functions in this website were well integrated |  |  |  |  |  |
| 6. I thought there was too much inconsistency in this website |  |  |  |  |  |
| 7. I would imagine that most people would learn to use this website very quickly |  |  |  |  |  |
| 8. I found this website very cumbersome/awkward to use |  |  |  |  |  |
| 9. I felt very confident using the website. |  |  |  |  |  |
| 10. I needed to learn a lot of things before I could get going with this website. |  |  |  |  |  |
