# Supplementary material for: The Healthy Hearts Project: Development and evaluation of a website for cardiovascular risk assessment and visualisation and self-management through healthy lifestyle goal-setting
Source: PLOS Digit Health. 2023 Nov 29;2(11):e0000395. doi: 10.1371/journal.pdig.0000395 (PMC10686463; doi:10.1371/journal.pdig.0000395)
Supplement: S5 Appendix — (DOCX) [file pdig.0000395.s006.docx]

**Appendix 5. Interheart Questionnaire with scoring**

| Question | Risk scoring |
| --- | --- |
|  |  |
| 1. What is your date of birth?  2. What is your sex? | 2 points for a man 55 years or older or a woman 65 years or older, 0 points otherwise |
| Diabetes  Do you have diabetes mellitus? | 6 points for Yes, 0 points for No or unsure |
| High blood pressure  Do you have high blood pressure? | 5 points for Yes, 0 points for No or unsure |
| Family History  Have either or both of your biological parents had a heart attack? | 4 points for Yes, 0 points for No |
| What is your waist measurement  What is your hip measurement | Waist:hip ratio  <0.873 0 points  0.873-0.963 2 points  >0.964 4 points |
| How often have you felt stress in the past year? | Never experienced stress 0 points  Some periods of stress 0 points  Several periods of stress 3 points  Permanent stress 3 points |
| How active are you during your leisure time? | Mainly sedentary 2 points  Mild exercise 2 points  Moderate exercise 0 points  Strenuous exercise 0 points |
| Do you eat salty foods or snacks one or more times a day? | Yes 1 point  No 0 points |
| Do you eat deep-fried food or snacks one or more times a day? | Yes 1 points  No 0 points |
| Do you eat fruit one or more times daily? | Yes 0 points  No 1 point |
| Do you eat vegetables one or more times daily? | Yes 0 points  No 1 point |
| Do you eat meat and/or poultry two or more times daily? | Yes 2 points  No 0 points |
