# Supplementary material for: The Healthy Hearts Project: Development and evaluation of a website for cardiovascular risk assessment and visualisation and self-management through healthy lifestyle goal-setting
Source: PLOS Digit Health. 2023 Nov 29;2(11):e0000395. doi: 10.1371/journal.pdig.0000395 (PMC10686463; doi:10.1371/journal.pdig.0000395)
Supplement: S6 Appendix — (DOCX) [file pdig.0000395.s007.docx]

**Appendix 6. International Physical Activity Questionnaire with scoring**

**Questions**

Q1. During the last 7 days on how many days did you do vigorous physical activity?

Q2. How much time did you usually spend doing vigorous physical activity on one of those days?

Q3. During the last 7 days on how many days did you do moderate physical activity?

Q4. How much time did you usually spend doing moderate physical activity on one of those days?

Q5. During the last 7 days, on how many days did you walk for at least 10 minutes at a time?

Q6. How much time did you spend walking on one of those days?

Q7. During the last 7 days, how much time did you spend sitting on a week day?

**Scoring**

The questionnaire was scored both to give a classification of overall activity as LOW, MODERATE, or HIGH, and to give a score that was used to visually present activity level on a scale.

Classification of activity score is based on either minutes of moderate and vigorous activity per week or on total MET (metabolic equivalents) minutes.

Vigorous_activity_per_week = Q1 (days per week) X Q2 (minutes per day)

Moderate_activity_per_week = Q3 (days per week) X Q4 (minutes per day)

Walking_per_week = Q5 (days per week) X Q6 (minutes per day)

Total_minutes_moderate_activity_per_week = (Vigorous_activity_per_week X 2) + Moderate_activity_per_week + Walking_per_week

Total_MET_minutes_per_week = (Vigorous_activity_per_week X 8) + (Moderate_activity_per_week X 4) + (Walking_per_week X 3.3)

If Total_minutes_moderate_activity_per_week <150 then activity level is **LOW.**

If there is vigorous activity on 3 or more days per week and Total_MET_minutes_per_week is ≥1500 OR Total_MET_minutes_per_week is ≥ 3000 then activity level is **HIGH.**

If Total_minutes_moderate_activity_per_week ≥150 and participant does not meet criterion for HIGH activity then activity levels is **MODERATE.**

Point shown on sliding activity scale

Activity level is also represented by a point on a sliding scale. The top point of the scale is set to Total_MET_minutes_per_week = 5040 (based on an hour of moderate activity and an hour of vigorous activity every day) – scores higher than this are capped to this figure.

The scale has cut-points for moderate and vigorous activity, but these can be met by different Total_MET_minutes_per_week,

e.g. the threshold for moderate activity can be met by either 150 minutes of Moderate_activity_per_week = 600 Total_MET_minutes_per_week, or 150 minutes of Walking_per_week = 495 Total_MET_minutes_per_week

AND

Threshold for high activity can be met with either ≥1500 Total_MET_minutes_per_week with vigorous activity on 3+ days, OR ≥3000 Total_MET_minutes_per_week.

This is resolved by setting the cut-offs on the scale at 600 Total_MET_minutes_per_week for moderate activity and 3000 Total_MET_minutes_per_week for vigorous activity, and increasing the Total_MET_minutes_per_week to these cut-offs where the criterion for moderate/vigorous activity is met but the Total_MET_minutes_per_week are below 600/3000 respectively.
